# Supplementary material for: Differences in the Conformational Energy Landscape of CDK1 and CDK2 Suggest a Mechanism for Achieving Selective CDK Inhibition
Source: Cell Chem Biol. 2019 Jan 17;26(1):121–130.e5. doi: 10.1016/j.chembiol.2018.10.015 (PMC6344228; doi:10.1016/j.chembiol.2018.10.015)
Supplement: Document S1. Figures S1–S7 and Tables S1 and S2 [file mmc1.pdf]

Cell Chemical Biology, Volume *26*

Supplemental Information

Differences in the Conformational Energy Landscape of CDK1 and CDK2 Suggest a Mechanism for Achieving Selective CDK Inhibition

Daniel J. Wood, Svitlana Korolchuk, Natalie J. Tatum, Lan-Zhen Wang, Jane A. Endicott, Martin E.M. Noble, and Mathew P. Martin

Supplementary Information for

Differences in the conformational energy landscape of CDK1 and CDK2 suggest a mechanism for achieving selective CDK inhibition

Daniel J. Wood, Svitlana Korolchuk, Natalie J. Tatum, Lan-Zhen Wang, Jane A. Endicott, Martin E.M. Noble and Mathew P. Martin

Lead contact: Martin E.M. Noble
Email: martin.noble@ncl.ac.uk

This PDF file includes:

- Supplementary text
- Figs. S1 to S7
- Tables S1 to S3

Other supplementary materials for this manuscript include the following:

Table S3. Summary of data collection and structure refinement, related to Figures 3-5

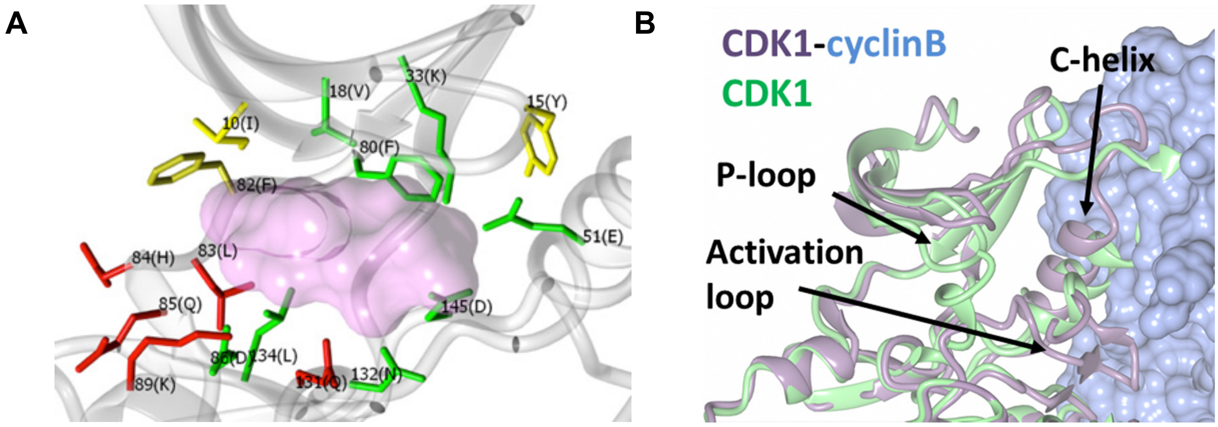

C

Residues numbers based on CDK2

	10	15	18	33	51	80	82	83	84	85	86	89	131	132	134	145
CDK1	I	Y	V	K	E	F	F	L	S	M	D	K	Q	N	L	D
CDK2	I	Y	V	K	E	F	F	L	H	Q	D	K	Q	N	L	D
CDK4	I	Y	V	K	E	F	H	V	D	Q	D	T	E	N	L	D
CDK6	I	Y	V	K	E	F	H	V	D	Q	D	T	Q	N	L	D
CDK8	V	Y	V	K	E	F	Y	A	E	H	D	H	A	N	L	D
CDK9	I	F	V	K	E	F	F	C	E	H	D	G	A	N	L	D
CDK12	I	Y	V	K	E	F	Y	M	D	H	D	G	S	N	L	D

Figure S1. The conserved nature of the CDK catalytic site. Related to Figure 1. (*A*) The active site of CDK2-cyclin A (PDB entry 1FIN) with key active site residues rendered as cylinders, ATP drawn as a transparent molecular surface coloured pink. The overall fold is drawn in white ribbon representation. The CDK2 active site residues are coloured by conservation (green, conserved; yellow, similar, red, non-conserved) between human CDK1, CDK2, CDK4, CDK6, CDK8, CDK9, and CDK12 (Uniprot entries P06493, P24941, P11802, Q00534, P49336, P50750 and Q9NYV4 respectively). At the protein sequence level, full length CDK1 and CDK2 share 64.5% sequence identity and 77.6 % similarity (EMBOSS Needle, EMBL-EBI). (*B*) CDK1 in the two structures (PDB entries 4YC3 (CDK1-cyclinB-Cks2, where CDK1 is represented in lilac ribbon and cyclin B in blue surface) and 4YC6 (CDK1-Cks1, where CDK1 is represented in green ribbon) differ through ordering of the N-terminal lobe and activation loop upon binding of cyclin

B. (*C*) Alignment of CDKs and their active site residues depicted in A), coloured by conservation (green, conserved; yellow, similar, red, non-conserved).

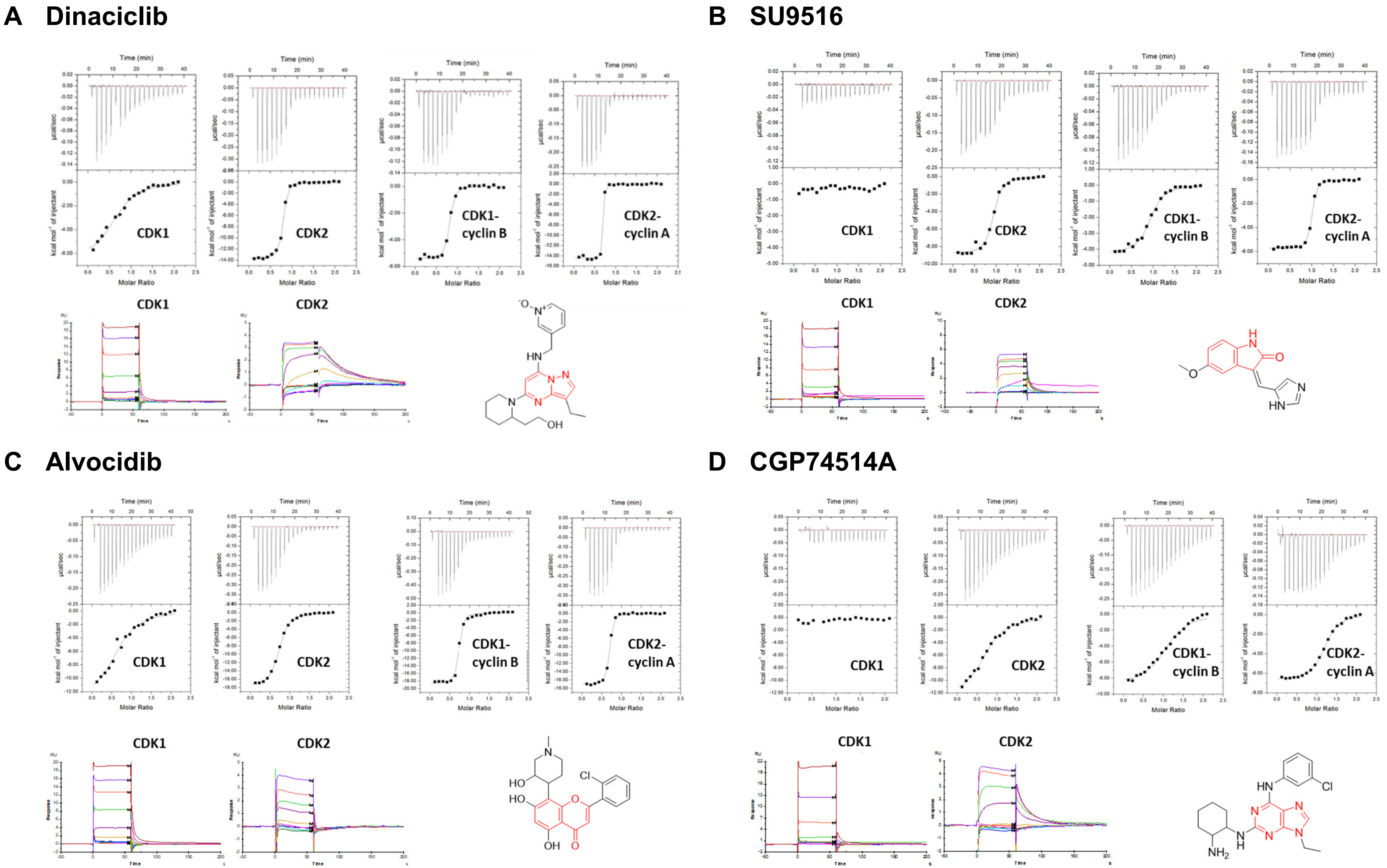
A Dinaciclib
B SU9516
C Alvocidib
D CGP74514A
Time (min)
µcal/sec
kcal mol-1 of injectant
Molar Ratio
CDK1
CDK2
CDK1-cyclin B
CDK2-cyclin A
Response
Time

Figure S2. ATP-competitive inhibitor binding to CDK1 and CDK2. Related to Table 1 and Figure 2. Isothermal titration calorimetry thermograms to assess binding of inhibitors Dinaciclib (*A*), SU9516 (*B*), Alvocidib (*C*), and CGP74514A (*D*) to CDK1, CDK2, CDK1-cyclin B, and CDK2- cyclin A. The inhibitors show reduced binding to monomeric CDK1 compared to CDK1-cyclin B (**Table 1**). Surface plasmon resonance sensorgrams to assess Dinaciclib (*A*), SU9516 (*B*), Alvocidib (*C*), and CGP74514A (*D*) binding to CDK1 and CDK2. Unphosphorylated CDK1 and CDK2 as GST fusions were immobilized on the SPR chip via anti-GST antibody coupling and inhibitor binding was assayed in duplicate over 11 (GST-CDK2) or 12 (GST-CDK1) concentrations as described in the Materials and Methods. Dissociation constants (**Table S2**) were derived by using the Biacore S200 Evaluation Software (GE Healthcare).

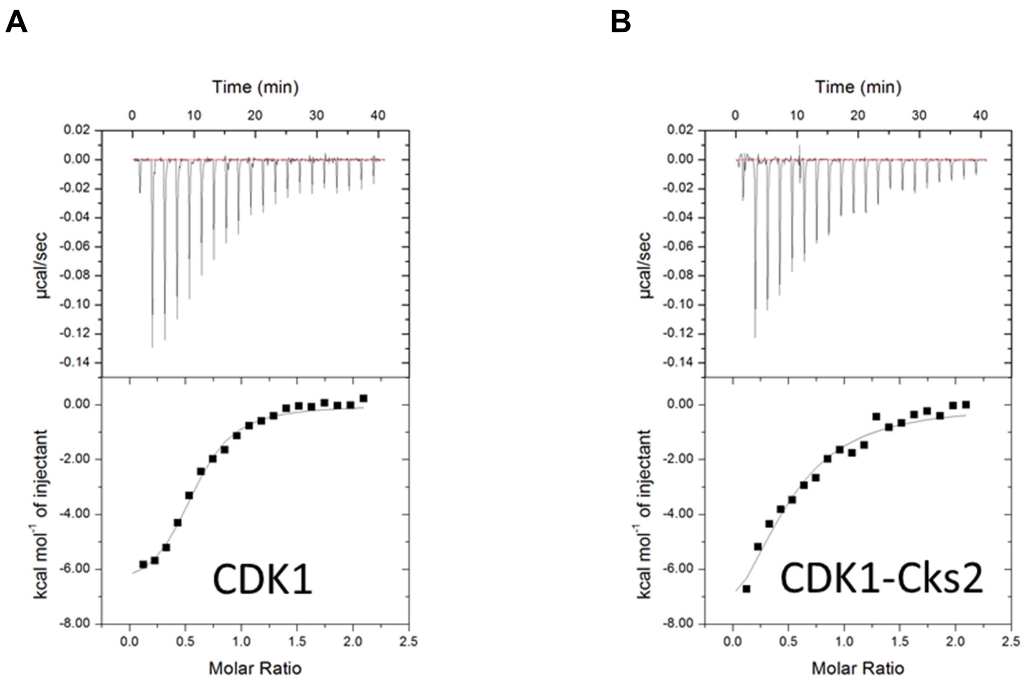

Figure S3. Comparison of the binding of Dinaciclib to CDK1 and CDK1-Cks2. Related to Figure 2. (*A*) Dinaciclib binding to CDK1. (This Figure panel is also included in Figure S1A). (*B*) Dinaciclib binding to CDK1-Cks2. This control was conducted in the presence of Cks2 so that any hypothesis inferred from the crystallographic observations would be supported. This titration experiment was performed as a singleton.

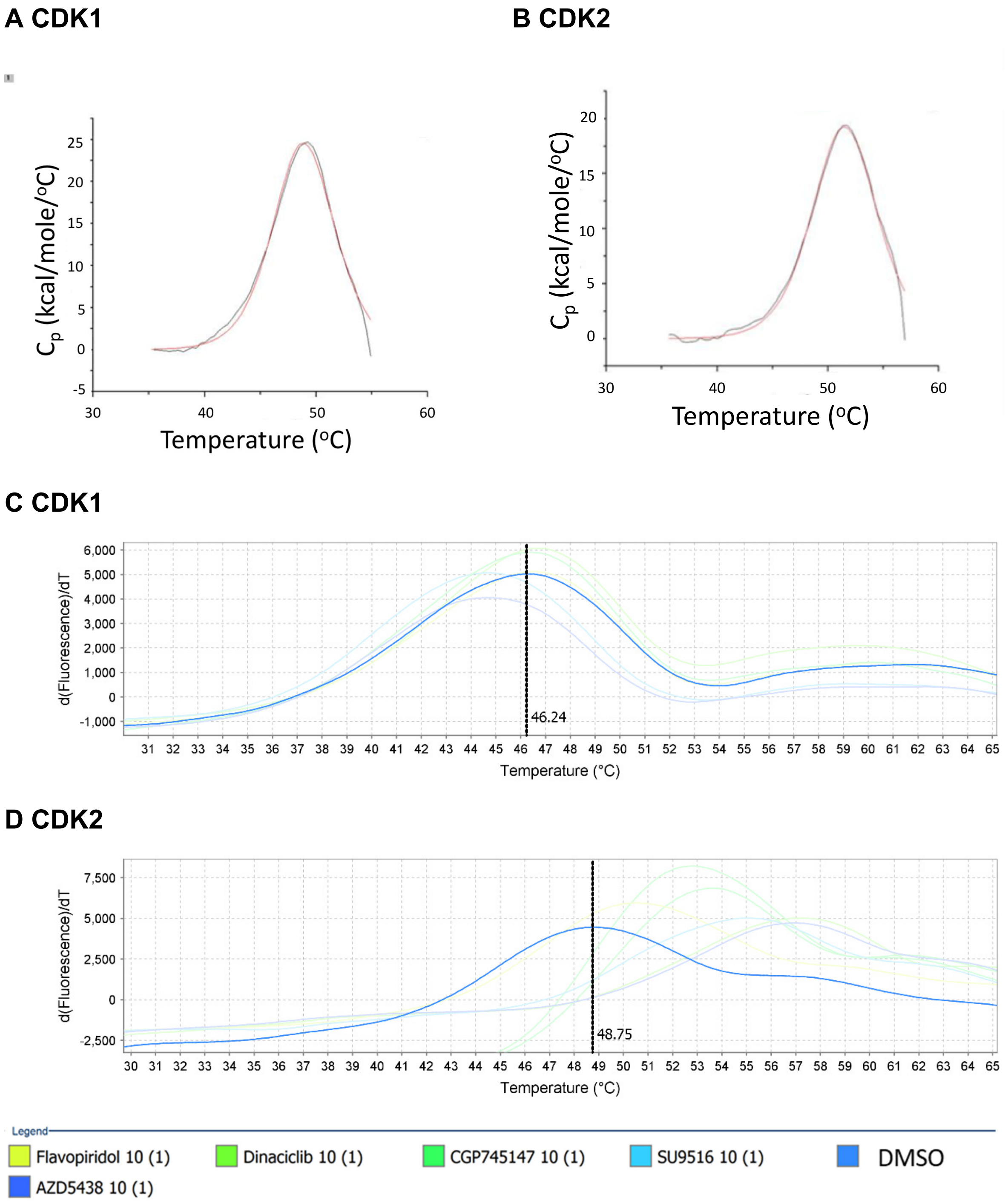

Figure S4. CDK1 and CDK2 stability assessed by differential scanning calorimetry and differential scanning fluorimetry Related to Table 2. (*A, B*) T_m determination. Differential

scanning calorimetry was used to determine T_m values for monomeric CDK1 and CDK2. (*C, D*) Differential scanning fluorimetry shows that ATP-competitive inhibitors have little effect on the stability of CDK1 (*C*) but stabilise CDK2 (*D*). Further experimental details are provided in the Materials and Methods.

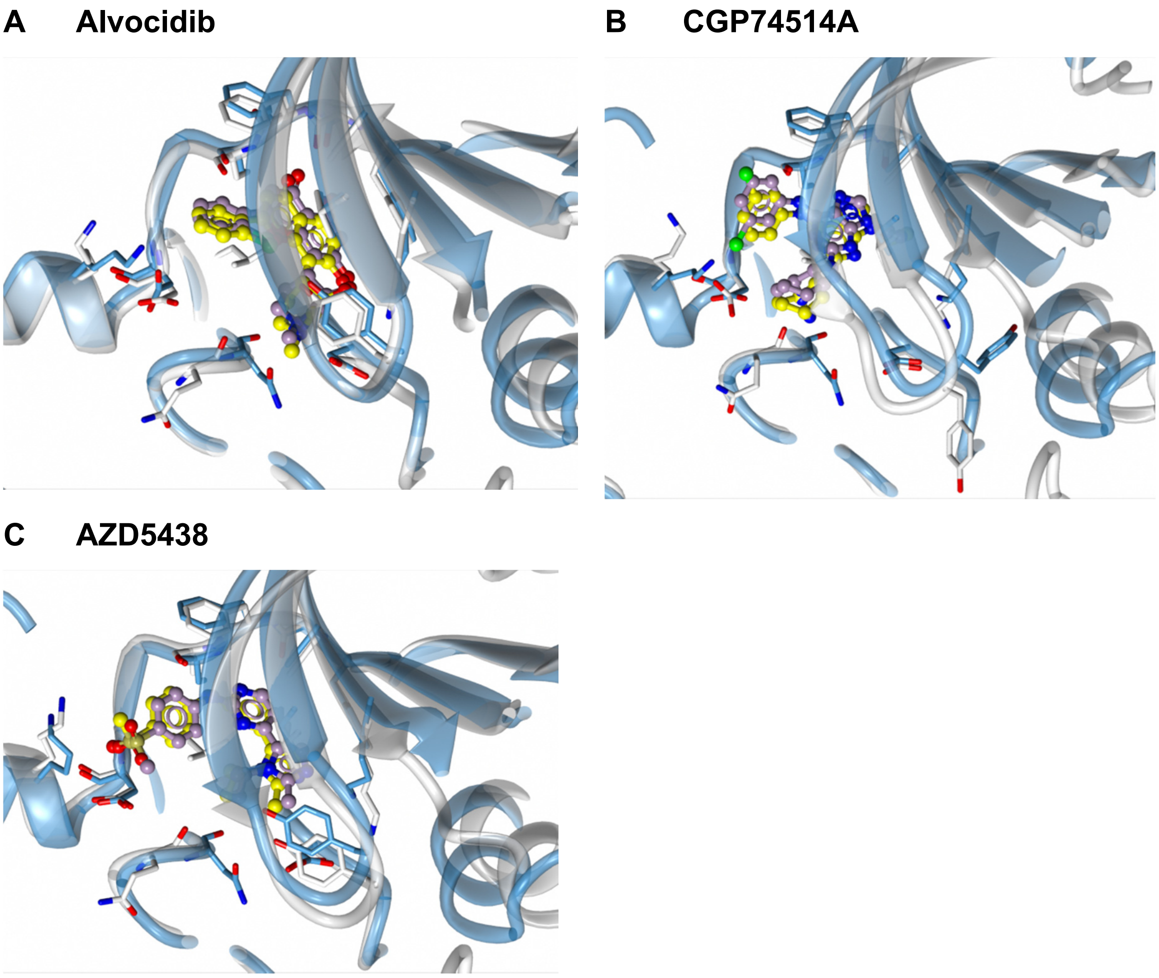

Figure S5. Comparison of ATP-competitive inhibitor binding to CDK1-cyclin B and CDK2-cyclin A. Related to Figure 3. Each of the three CDK1-cyclin B-Cks2-inhibitor co-complex structures presented in Figure 3 is overlaid with the corresponding CDK2-cyclin A-inhibitor co-complex structure. (*A*) Alvocidib, (*B*) CGP74514a and (*C*) AZD5438. The view in each case is identical to the view shown in Figure 3. CDK1-cyclin B is shown in white ribbon and cylinder with the inhibitor carbon atoms coloured yellow. CDK2-cyclin A is drawn in light blue with inhibitor carbon atoms coloured lilac.

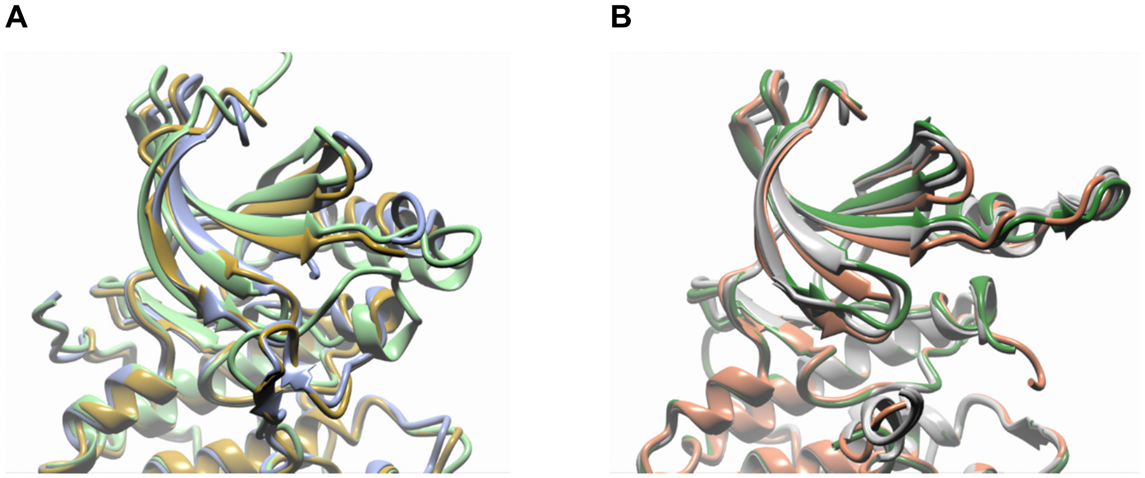

Figure S6. Comparison of cyclin-free CDK2 and CDK1 structural poses. Related to Figure 2-4 (*A*) Showing the flexibility of Cyclin-free CDK2 within the $P2_12_12_1$ crystal form; apo CDK2 (1HCK – blue), cyclin-free CDK2 bound to ANS (3PXZ - green), and the alternative lattice formed through binding of Cks1 (1BUH – gold) suggesting that this crystal form is capable of accommodating significantly different kinase conformations. (*B*) Superimposition of the 4 ASU copies of cyclin-free CDK1 (4YC6 - grey) with the two alternative lattices formed through association with dinaciclib (6GU6 - coral) and AZD5438 (6GU7 - green).

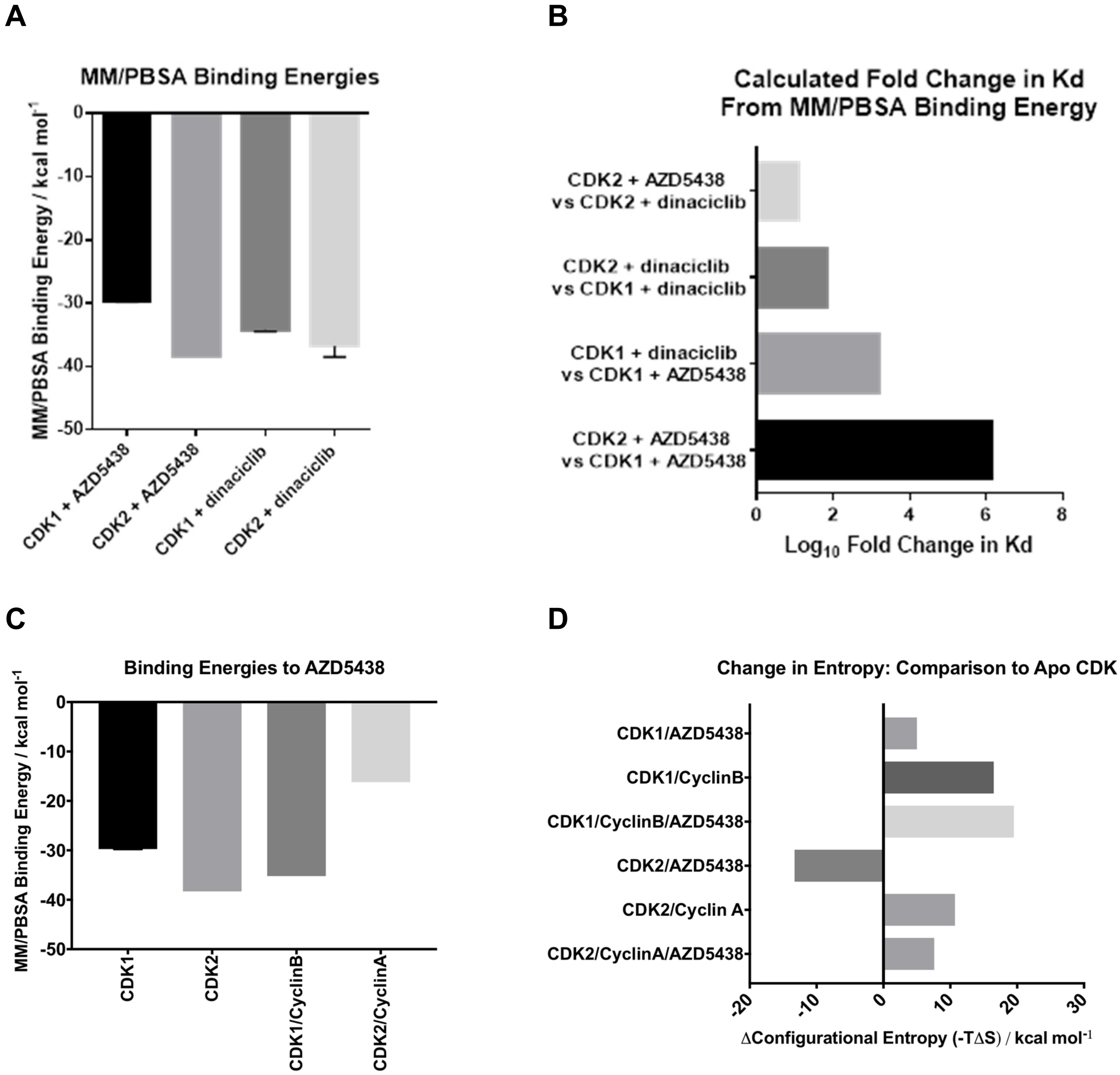

Figure S7. Simulations binding data for AZD5438. Related to Figure 2. In silico molecular dynamics simulations and MM/PBSA calculations against cyclin-free CDK1 and CDK2 with AZD5438 and Dinaciclib (A) and calculated fold change in Kd (B). Simulation analysis for monomeric and complex binding to AZD5438: binding energies derived from molecular dynamics simulations (C), and calculated change in configurational entropy in the CDK component of protein, ligand, and protein-ligand complexes (D).

SUPPLEMENTAL TABLES

	Published measurements (nM)		Lab measurements (nM)		Method	Reference
	CDK1B	CDK2A	CDK1B	CDK2A		
Dinaciclib	3/3/3/3	1/1/1/1	23 (12.8)	3.5 (1.9)	γ^{33}P-ATP streptavidin coated bead-based assay (biotinylated peptide of Histone H1)	(Whittaker et al., 2017), (Asghar et al., 2015), (Mariaule and Belmont, 2014), (Parry et al., 2010)
AZD5438	16/16	3/45	12 (6.7)	7.4 (4.1)	Scintillation proximity assay	(Whittaker et al., 2017), (Byth et al., 2009)
Alvocidib	27/30/27/ 30/30	405/170/40 5/100 70	8.8 (4.9)	26 (14.4)	Radiolabelled γ^{33}P-ATP kinase assay	(Whittaker et al., 2017),(Asghar et al., 2015),(Byth et al., 2009), (Mariaule and Belmont, 2014), (Kim et al., 2000)
CGP74514A	25/25	n/a	833 (463)	33 (18.3)	Radiolabelled γ33P-ATP kinase assay	(Imbach et al., 1999)
SU9516	40/40	22/22	186 (103)	38 (21)	Radiolabelled γ^{33}P-ATP kinase assay	(Lane et al., 2001)

Table S1. Inhibitor IC_{50} values. Related to Figure 2. IC_{50} values for each of the five inhibitors against CDK1-cyclin B and CDK2-cyclin A were determined using an ADPGlo^{TM} assay format (Brown et al., 2015) and compared to literature values. K_i values denoted in parenthesis

A

	AZD5438 vs CDK1-cyclin B	AZD5438 vs CDK2-cyclin A	AZD5438 vs CDK1	AZD5438 vs CDK2
**N (sites)**	0.58	0.68	0.65	0.70
**K_a (M^{-1})**	$1.76 \times 10^{7} \pm 1.83 \times 10^{6}$	$3.95 \times 10^{8} \pm 1.38 \times 10^{8}$	$8.42 \times 10^{5} \pm 1.54 \times 10^{5}$	$3.38 \times 10^{7} \pm 4.01 \times 10^{6}$
**K_d (nM)**	57.0 ± 5.9	2.50 ± 0.88	1200 ± 220	29.5 ± 3.5
**ΔH (kcal/mol)**	-12.80 ± 0.11	-13.40 ± 0.06	-6.44 ± 0.38	-16.20 ± 0.11
**ΔS (cal/mol/deg)**	-9.1	-4.7	5.9	-19.1

	CGP74514A vs CDK1-cyclin B	CGP74514A vs CDK2-cyclin A	CGP74514A vs CDK1	CGP74514A vs CDK2
**N (sites)**	1.01	1.01	No fitting	1.17
**K_a (M^{-1})**	$1.11 \times 10^{6} \pm 3.09 \times 10^{5}$	$4.74 \times 10^{6} \pm 4.69 \times 10^{5}$		$1.43 \times 10^{6} \pm 2.72 \times 10^{5}$
**K_d (nM)**	900 ± 250	210 ± 21	>20000	700 ± 130
**ΔH (kcal/mol)**	-6.09 ± 0.34	-6.09 ± 0.07		-4.60 ± 0.14
**ΔS (cal/mol/deg)**	7.6	10.5		13.0

	Dinaciclib vs CDK1-cyclin B	Dinaciclib vs CDK2-cyclin A	Dinaciclib vs CDK1	Dinaciclib vs CDK2
**N (sites)**	0.71	0.70	0.57	0.82
**K_a (M^{-1})**	$3.4 \times 10^{7} \pm 5.64 \times 10^{6}$	$3.19 \times 10^{8} \pm 1.55 \times 10^{8}$	$1.41 \times 10^{6} \pm 2.34 \times 10^{5}$	$1.82 \times 10^{7} \pm 3.95 \times 10^{6}$
**K_d (nM)**	29.4 ± 4.9	3.1 ± 1.5	709 ± 120	55 ± 12
**ΔH (kcal/mol)**	-7.10 ± 0.07	-12.40 ± 0.08	-6.9 ± 0.3	-13.30 ± 0.21
**ΔS (cal/mol/deg)**	11.0	-2.1	5.3	-10.8

	SU9516 vs CDK1-cyclin B	SU9516 vs CDK2-cyclin A	SU9516 vs CDK1	SU9516 vs CDK2
**N (sites)**	0.97	0.98	No fitting	0.95
**K_a (M^{-1})**	$4.68 \times 10^{6} \pm 4.11 \times 10^{5}$	$3.28 \times 10^{7} \pm 3.06 \times 10^{6}$		$1.02 \times 10^{7} \pm 1.57 \times 10^{6}$
**K_d (nM)**	214 ± 19	30.5 ± 2.8	>20000	98 ± 15
**ΔH (kcal/mol)**	-4.39 ± 0.04	-5.02 ± 0.03		-6.28 ± 0.08
**ΔS (cal/mol/deg)**	16.0	17.8		11.4

	Alvocidib vs CDK1-cyclin B	Alvocidib vs CDK2-cyclin A	Alvocidib vs CDK1	Alvocidib vs CDK2
**N (sites)**	0.71	0.80	0.61	0.82
**K_a (M^{-1})**	$4.51 \times 10^{7} \pm 8.5 \times 10^{6}$	$5.08 \times 10^{7} \pm 1.04 \times 10^{7}$	$5.15 \times 10^{5} \pm 7.89 \times 10^{4}$	$4.6 \times 10^{6} \pm 5.01 \times 10^{5}$
**K_d (nM)**	22.2 ± 4.2	20 ± 4	1900 ± 300	217.0 ± 23.7
**ΔH (kcal/mol)**	-16.60 ± 0.16	-15.10 ± 0.14	-10.70 ± 0.74	-13.80 ± 0.18
**ΔS (cal/mol/deg)**	-19.8	-14.5	-9.3	-14.9

B

	AZD5438 vs CDK1-cyclin B	AZD5438 vs CDK2-cyclin A	AZD5438 vs CDK1	AZD5438 vs CDK2
**N (sites)**	0.58	0.56	0.65	0.56
**K_a (M^{-1})**	$1.20 \times 10^{7} \pm 3.77 \times 10^{6}$	$2.22 \times 10^{8} \pm 4.73 \times 10^{7}$	$1.32 \times 10^{5} \pm 2.53 \times 10^{4}$	$4.28 \times 10^{7} \pm 3.71 \times 10^{6}$
**K_d (nM)**	83 ± 26	4.5 ± 1.0	7600 ± 1500	23 ± 2
**ΔH (kcal/mol)**	-14.13 ± 0.42	-16 ± 0.08	-18.8 ± 3.9	-19.9 ± 0.1
**ΔS (cal/mol/deg)**	-14.2	-14.6	-38.5	-30.7

	CGP74514A vs CDK1-cyclin B	CGP74514A vs CDK2-cyclin A	CGP74514A vs CDK1	CGP74514A vs CDK2
**N (sites)**	0.73	1.18	No fitting	1.09
**K_a (M^{-1})**	$9.82 \times 10^{5} \pm 1.31 \times 10^{5}$	$4.21 \times 10^{6} \pm 3.34 \times 10^{5}$		$1.37 \times 10^{6} \pm 2.27 \times 10^{5}$
**K_d (nM)**	1000 ± 140	240 ± 19	>20000	730 ± 120
**ΔH (kcal/mol)**	-12.6 ± 0.5	-6.70 ± 0.06		-8.73 ± 0.25
**ΔS (cal/mol/deg)**	-14.1	8.2		-0.7

	Dinaciclib vs CDK1-cyclin B	Dinaciclib vs CDK2-cyclin A	Dinaciclib vs CDK1	Dinaciclib vs CDK2
**N (sites)**	0.77	0.65	0.67	0.75
**K_a (M^{-1})**	$2.93 \times 10^{7} \pm 4.71 \times 10^{6}$	$9.93 \times 10^{8} \pm 3.73 \times 10^{8}$	$8.44 \times 10^{5} \pm 1.68 \times 10^{5}$	$3.73 \times 10^{7} \pm 3.74 \times 10^{6}$
**K_d (nM)**	34.0 ± 5.5	1.0 ± 0.4	1200 ± 240	27 ± 3
**ΔH (kcal/mol)**	-5.40 ± 0.05	-14.40 ± 0.09	-6.56 ± 0.39	-13.70 ± 0.08
**ΔS (cal/mol/deg)**	16.4	-6.43	5.5	-10.4

	SU9516 vs CDK1-cyclin B	SU9516 vs CDK2-cyclin A	SU9516 vs CDK1	SU9516 vs CDK2
**N (sites)**	0.91	0.96	No fitting	0.90
**K_a (M^{-1})**	$3.94 \times 10^{6} \pm 5.53 \times 10^{5}$	$4.88 \times 10^{7} \pm 4.1 \times 10^{6}$		$1.02 \times 10^{7} \pm 1.77 \times 10^{6}$
**K_d (nM)**	254 ± 36	21.0 ± 1.7	>20000	98 ± 17
**ΔH (kcal/mol)**	-4.24 ± 0.07	-5.72 ± 0.02		-8.81 ± 0.13
**ΔS (cal/mol/deg)**	16.2	16.3		3.0

	Alvocidib vs CDK1-cyclin B	Alvocidib vs CDK2-cyclin A	Alvocidib vs CDK1	Alvocidib vs CDK2
**N (sites)**	0.69	0.65	0.68	0.70
**K_a (M^{-1})**	$3.01 \times 10^{7} \pm 6.5 \times 10^{6}$	$4 \times 10^{7} \pm 3.1 \times 10^{6}$	$7.44 \times 10^{5} \pm 1.92 \times 10^{5}$	$4.63 \times 10^{6} \pm 2.08 \times 10^{5}$
**K_d (nM)**	33.0 ± 7.2	25 ± 2	1300 ± 350	220.0 ± 9.7
**ΔH (kcal/mol)**	-18.60 ± 0.24	-17.10 ± 0.08	-13.2 ± 1.1	-17.8 ± 0.1
**ΔS (cal/mol/deg)**	-27.3	-21.5	-16.6	-28.2

Table S2. ITC experimental data for inhibitor binding to cyclin-free CDK1, CDK2 and their respective complexes with cognate cyclin partners. Related to Figure 2. Experiments were carried out in duplicate biological replicates shown in panels A and B.

Table S3. Summary of data collection and structure refinement. related to Figures 3-5

See associated Excel file: ‘TableS3CrystallographyData.xlsx’
