# Supplementary material for: Differences in the Conformational Energy Landscape of CDK1 and CDK2 Suggest a Mechanism for Achieving Selective CDK Inhibition
Source: Cell Chem Biol. 2019 Jan 17;26(1):121–130.e5. doi: 10.1016/j.chembiol.2018.10.015 (PMC6344228; doi:10.1016/j.chembiol.2018.10.015)
Supplement: Document S2. Article plus Supplemental Information [file mmc3.pdf]

Article

Cell Chemical Biology

Differences in the Conformational Energy Landscape of CDK1 and CDK2 Suggest a Mechanism for Achieving Selective CDK Inhibition

Graphical Abstract

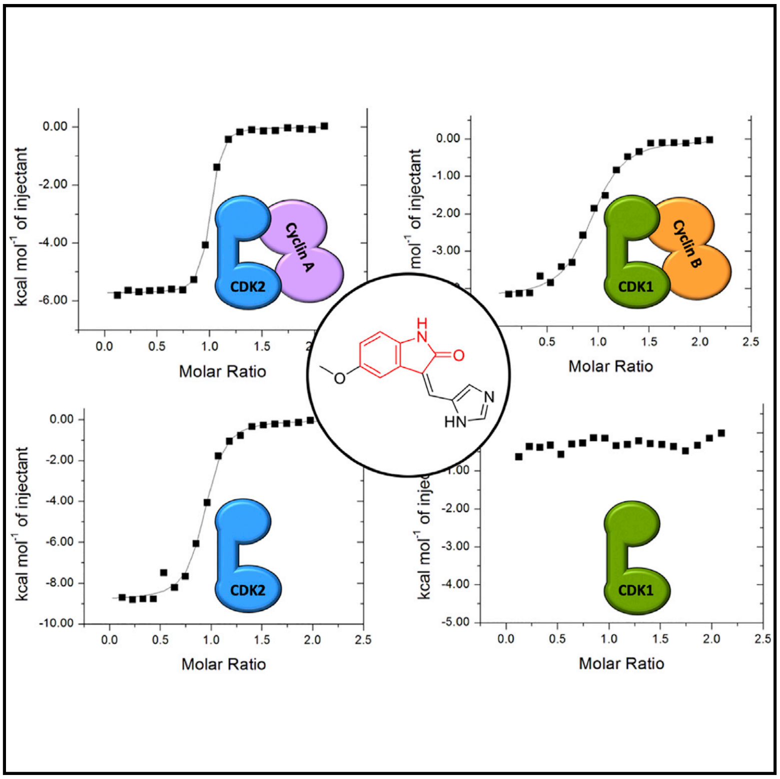

Authors

Daniel J. Wood, Svitlana Korolchuk, Natalie J. Tatum, Lan-Zhen Wang, Jane A. Endicott, Martin E.M. Noble, Mathew P. Martin

Correspondence

martin.noble@ncl.ac.uk (M.E.M.N.), mathew.martin@ncl.ac.uk (M.P.M.)

In Brief

Current CDK2 small-molecule inhibitors also inhibit CDK1, generating a toxicity liability. Biophysical measurements demonstrate that structurally diverse ATP-competitive inhibitors discriminate between monomeric CDK1 and CDK2. The unusual properties of monomeric CDK1 might be exploited to differentiate CDK1 from other CDKs in future cancer therapeutic design.

Highlights

- Demonstration that ATP-competitive inhibitors distinguish cyclin-free CDK1 from CDK2
- Determination of the first cyclin-free CDK1-ATP inhibitor complex structures
- Targeting cyclin-free CDKs may offer a clear selectivity window over CDK1

Wood et al., 2019, Cell Chemical Biology *26*, 121–130
January 17, 2019 © 2018 The Authors. Published by Elsevier Ltd.
https://doi.org/10.1016/j.chembiol.2018.10.015

CellPress

Cell Chemical Biology
Article

CellPress

Differences in the Conformational Energy Landscape of CDK1 and CDK2 Suggest a Mechanism for Achieving Selective CDK Inhibition

Daniel J. Wood,[1,2] Svitlana Korolchuk,[1,2] Natalie J. Tatum,[1] Lan-Zhen Wang,[1] Jane A. Endicott,[1] Martin E.M. Noble,[1,3,*] and Mathew P. Martin[1,*]
[1]Newcastle Cancer Centre, Northern Institute for Cancer Research, Medical School, Newcastle University, Paul O'Gorman Building, Framlington Place, Newcastle upon Tyne NE2 4HH, UK
[2]These authors contributed equally
[3]Lead contact
*Correspondence: martin.noble@ncl.ac.uk (M.E.M.N.), mathew.martin@ncl.ac.uk (M.P.M.)
https://doi.org/10.1016/j.chembiol.2018.10.015

SUMMARY

Dysregulation of the cell cycle characterizes many cancer subtypes, providing a rationale for developing cyclin-dependent kinase (CDK) inhibitors. Potent CDK2 inhibitors might target certain cancers in which *CCNE1* is amplified. However, current CDK2 inhibitors also inhibit CDK1, generating a toxicity liability. We have used biophysical measurements and X-ray crystallography to investigate the ATP-competitive inhibitor binding properties of cyclin-free and cyclin-bound CDK1 and CDK2. We show that these kinases can readily be distinguished by such inhibitors when cyclin-free, but not when cyclin-bound. The basis for this discrimination is unclear from either inspection or molecular dynamics simulation of ligand-bound CDKs, but is reflected in the contacts made between the kinase N- and C-lobes. We conclude that there is a subtle but profound difference between the conformational energy landscapes of cyclin-free CDK1 and CDK2. The unusual properties of CDK1 might be exploited to differentiate CDK1 from other CDKs in future cancer therapeutic design.

INTRODUCTION

Members of the cyclin-dependent protein kinase (CDK) family have diverse cellular roles that include regulation of the cell cycle and transcription and, in certain cell types, differentiation (Lim and Kaldis, 2013; Malumbres, 2014; Morgan, 2007). Dysregulation of CDK activity is frequently associated with inappropriate cell-cycle progression and, as a result, various members of the family have been pursued as targets for anti-cancer drug design (Kim et al., 2013; Toogood et al., 2005; Whittaker et al., 2017). *CCNE1* encodes cyclin E, which binds to CDK2 to drive cells through the G1/S cell-cycle transition (Koff et al., 1991; Malumbres, 2014; Morgan, 2007). The results observed when CDK2 levels are genetically suppressed indicate that its function is not essential for mitosis in normal tissue development and homeostasis (Berthet et al., 2003). In contrast, tumor cells in which *CCNE1* is amplified are critically dependent on CDK2 and cyclin E for survival (Etemadmoghadam et al., 2013a, 2013b). *CCNE1* amplification or cyclin E1 overexpression has also been described in a number of other cancers including osteosarcoma (Lockwood et al., 2011), breast (Karakas et al., 2016), and non-small-cell lung cancer (Freemantle and Dmitrovsky, 2010). Such "oncogene-addiction" to cyclin E occurs in a significant cohort of high-grade serous ovarian cancer (HGSOC) patients and confers a particularly poor outcome to current therapy (Kanska et al., 2016; Kroeger and Drapkin, 2017; Patch et al., 2015). These findings suggest that a CDK2-selective inhibitor could be clinically beneficial in these cancer subtypes.

CDKs share a conserved protein kinase domain that comprises a smaller N-terminal lobe linked through a hinge to a larger C-terminal fold (Endicott et al., 2012). Cyclin-free CDKs are inactive because the C helix, the activation segment (the sequence between the conserved DFG and APE motifs, single-letter amino acid code, CDK1 residues 146–173), and the P loop (the glycine-rich phosphate binding sequence, CDK1 residues 11–17) are inappropriately disposed to promote catalysis (De Bondt et al., 1993; Russo et al., 1996). The C helix, which lies at the back of the active site cleft, is rotated out of the fold, its position dictated in part by the DFG sequence adopting a short α-helical structure. The activation segment is flexible, illustrated in a number of cyclin-free structures determined by X-ray crystallography, where it is either not visible or adopts alternative structures that fold toward the P loop (Brown et al., 2015; Martin et al., 2017).

All CDKs require binding to a cognate cyclin partner to remould the kinase fold from an inactive conformation to one capable of phospho-transfer. For example, CDK1 and CDK2 are partnered by cyclins A and B, and A and E, respectively (Malumbres, 2014). Cyclins bind to the C helix, and remodel the CDKs so that residues of the C helix and the DFG motif are aligned for catalysis (Echalier et al., 2010; Morgan, 2007). Cyclin binding also restructures the activation segment and, generally, this re-organization is accompanied by phosphorylation of the activation segment by the CDK-activating kinase (Desai et al., 1995; Merrick et al., 2008). These structural changes, local to the active site, reflect a global change in the relative positions of the kinase N- and C-lobes. However, structural studies

This is an open access article under the CC BY license (http://creativecommons.org/licenses/by/4.0/).

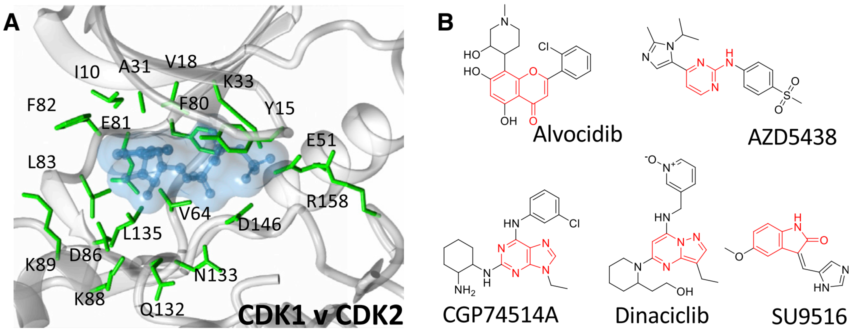

Figure 1. ATP-Competitive Inhibitor Binding to the CDK Active Site

(A) Active site sequence conservation between CDK1 and CDK2. The CDK2 structure bound to ATP is drawn (PDB: 1HCK). Residues within 4.5 Å of ATP are drawn in ball and stick mode in green and are identical between CDK1 and CDK2. The molecular surface of the bound ATP is rendered as a transparent blue surface to indicate the volume occupied within the active site.

(B) CDK inhibitors Alvocidib, AZD5438, CGP74514A, Dinaciclib, and SU9516 used in this study. The pharmacophore scaffolds are highlighted in red.

suggest that, at least for CDKs 2 and 4, some structural details of the catalytically competent Michaelis complex only form upon binding of both ATP and peptide substrates.

The first generation of non-selective CDK inhibitors that were evaluated in the clinic showed limited therapeutic benefit due to dose-limiting toxicities (Whittaker et al., 2017). The essential role of CDK1 in the normal cell cycle suggests that some of these toxicities might be avoided by excluding CDK1 from the inhibitory profile of drugs that target CDKs, raising the question of how CDK1 discrimination might be achieved. Structures of CDK-cyclin complexes bound to ATP-competitive inhibitors reveal how differences in amino acid composition and in the conformation and malleability of sequences in and around the active site can identify potent and selective molecules (Martin et al., 2017; Whittaker et al., 2017). More dispersed changes that reflect the plasticity of the ATP binding site have also been hypothesized to generate inter-CDK selectivity (Coxon et al., 2017; Hole et al., 2013; Shao et al., 2013). However, the residues that contact ATP in a CDK2-cyclin A-AMPPNP-peptide complex are identical in CDK1 (Brown et al., 1999), while cell-active CDK2 inhibitor series appear not to be sufficiently selective to exert *CCNE1* amplicon-dependent cell growth inhibition in models of HGSOC (Au-Yeung et al., 2017).

An alternative route to discriminate between CDK2 and CDK1 would be to target the cyclin-free structures of the enzymes. The structure of CDK1 appears to differ from that of CDK2 in terms of the disposition of N- and C-terminal lobes, while it also appears more mobile and malleable (Brown et al., 2015). Inhibitors have been reported that exploit the conformational flexibility of cyclin-free CDK2 to target pockets that stabilize conformations incompatible with cyclin association (Alexander et al., 2015; Betzi et al., 2011; Deng et al., 2014). Taken together, these observations suggest that targeting cyclin-free CDK structures might provide a selectivity window between closely related CDK family members.

We have applied a range of biophysical techniques to discover that ATP-competitive inhibitors bind with a surprisingly low affinity to cyclin-free CDK1 as compared with CDK2. Binding of CDK1 and CDK2 to their respective cognate partners, cyclin B and cyclin A, largely removes this difference, so that the cyclin-bound forms share much more similar inhibitor binding affinities. We conclude that cyclin-free CDK1 binds inhibitors weakly because its conformational energy landscape offers stable states that are incompetent to bind inhibitors, thereby moderating the free energy of binding that derives from protein-ligand interactions. Our results demonstrate that small-molecule inhibitors that probe the ATP binding site can distinguish cyclin-free CDK1 and CDK2, thereby offering a structural mechanism to enable development of potent and selective CDK2 inhibitors.

RESULTS

ATP-Competitive Inhibitors Discriminate between Cyclin-free CDK1 and CDK2

Avoiding the inhibition of CDK1 in a multi-CDK or single-CDK inhibition strategy is complicated by the high degree of active site sequence identity within the CDK family, illustrated for CDKs 1 and 2 in Figure 1A (Figure S1). To explore the potential for ATP-competitive inhibitors to discriminate between CDK1 and CDK2, five ATP-competitive CDK inhibitors (Figure 1B) were selected on the basis of their diverse pharmacophores and submicromolar half maximal inhibitory concentration (IC_{50}) values toward CDK2-cyclin A and CDK1-cyclin B (Table S1). The set includes Dinaciclib (Parry et al., 2010), AZD5438 (Byth et al., 2009), Alvocidib (Flavopiridol) (Carlson et al., 1996), SU9516 (Lane et al., 2001), and CGP74514A (Imbach et al., 1999). To confirm previously reported activities, these inhibitors were first tested for their ability to bind to CDK1-cyclin B and CDK2-cyclin A using isothermal titration calorimetry (ITC) (Tables 1 and S2). The inhibitor binding isotherms correlate with the reported IC_{50} data and our independent IC_{50} measurements (Table S1). AZD5438 (Figure 2A), Dinaciclib (Figure S2A), and SU9516 (Figure S2B) bind with low-nanomolar affinity toward CDK2-cyclin A and show up to 16-fold selectivity for CDK2-cyclin A over CDK1-cyclin B (Table 1). Reflecting its pan-CDK inhibition profile, Alvocidib is equipotent toward CDK1-cyclin B and CDK2-cyclin A (Figure S2C; Table 1). The purine-based CDK1-cyclin B-selective inhibitor CGP74514A is a less-potent CDK inhibitor (Table S1), and binds with reduced affinity to CDK1-cyclin B and to CDK2-cyclin A under these experimental conditions (Figure S2D; Table 1). Taken together these results show that the affinities and potencies of a set of structurally diverse ATP-competitive CDK inhibitors toward CDK2-cyclin A correlate with their values toward CDK1-cyclin B.

We next investigated the ability of each inhibitor to bind to cyclin-free phosphorylated CDK1 and CDK2. A range of inhibitor binding isotherms were measured for CDK1 (Table 1). Dinaciclib proved to be the tightest binding inhibitor, with a measured K_d of 0.96 μM (Figure S2A), while the binding of SU9516 did not reach

Table 1. Inhibitor Binding to CDK1-Cyclin B, CDK2-Cyclin A, CDK1, and CDK2

	ITC K_d (nM)			
	CDK1B	CDK2A	CDK1	CDK2
Dinaciclib	32 ± 2	2 ± 1	955 ± 246	41 ± 14
AZD5438	70 ± 13	4 ± 1	4,400 ± 3,200	26 ± 3
Alvocidib	28 ± 6	23 ± 3	1,600 ± 300	217 ± 1
CGP74514A	950 ± 50	224 ± 14	>20,000	715 ± 15
SU9516	234 ± 20	25 ± 5	>20,000	98 ± 1

Inhibitor binding was measured by isothermal titration calorimetry (ITC). ITC titrations were carried out using phosphorylated monomeric CDK1 and CDK2, and CDK1-cyclin B (CDK1B) and CDK2-cyclin A (CDK2A) complexes. Experiments were carried out with two biological replicates (i.e., with n = 2), and values are quoted ± SD. Full results for both replicates are shown in Table S2.

saturation under these experimental conditions, implying a K_d greater than 20 μM (Figure S2B). In contrast, both Dinaciclib and SU9516 retained their ability to bind tightly to cyclin-free CDK2, returning K_d values of 41 and 98 nM, respectively. The same pattern is seen for comparative binding of AZD5438 (Figure 2B), Alvocidib (Figure S2C), and CGP74514A (Figure S2D) to cyclin-free CDK1 and CDK2. Taken together the ITC results reveal that ATP-competitive inhibitors show substantially weaker binding to cyclin-free CDK1 than to CDK2 (Figure 2D). Moreover, a significantly reduced binding affinity is observed for inhibitor binding to cyclin-free CDK1 in comparison with the cyclin B bound complex (Table 1).

Using AZD5438 ITC isotherms we were able to calculate the relative enthalpic and entropic contributions involved in inhibitor binding to the different kinases in cyclin-free and cyclin-bound states (Figure 2E). From this analysis, it appears that binding to CDK1 and CDK2, in both cyclin-bound and cyclin-free forms, is enthalpically favorable but entropically unfavorable under the ITC conditions. However, binding of the inhibitor to either of the cyclin-bound CDKs is less entropically unfavorable than binding to the corresponding cyclin-free CDK, suggesting that cyclin binding pre-organizes the CDK moiety so that inhibitor binding carries a lower entropic penalty. Inhibitor binding to cyclin-free CDK1 carries a similar entropic penalty to that associated with binding to cyclin-free CDK2, so that the apparent difference in affinity cannot simply be attributed to higher conformational flexibility in apo cyclin-free CDK1.

We next employed surface plasmon resonance (SPR) as an orthogonal technique to measure inhibitor binding to cyclin-free CDK1 and CDK2 (Table 2). Glutathione-S-transferase (GST) fusions of CDK1 and CDK2 were immobilized on a chip coated with an anti-GST antibody and each inhibitor was flowed over as the analyte. This study fully corroborated the results of the ITC. AZD5438 (Figure 2C), Dinaciclib, SU9516, Alvocidib, and CGP74514A (Figures S2A–S2D) all bound tightly to CDK2 with K_d values of 43, 78, 56, 650, and 312 nM, respectively. However, the inhibitor affinities (K_d) toward immobilized CDK1 were all greater than 1 μM (Figures 2C and S2A–S2D).

Finally, we assessed the stability of cyclin-free forms of CDK1 and CDK2 using differential scanning calorimetry, and their further stabilization upon inhibitor binding using differential scanning fluorimetry. cyclin-free CDK1 has a lower melting temperature than does CDK2 (48.9°C ± 0.04°C versus 51.52°C ± 0.05°C, Figures S4A and S4B) and, as expected from the SPR and ITC experiments, inhibitor binding stabilized CDK2 to thermal denaturation but had little effect on CDK1 (Table 2; Figures S4C and S4D). In summary, our biophysical analyses demonstrate that ATP-competitive inhibitors bind tightly to cyclin-bound CDK1 and CDK2 and to cyclin-free CDK2, but have much reduced affinity for cyclin-free CDK1.

The Binding Mode of ATP-Competitive Inhibitors Is Conserved between CDK1-Cyclin B and CDK2-Cyclin A

To elaborate the molecular interactions that mediate the different binding affinities of inhibitors for cyclin-free and cyclin-bound forms of CDK1 and CDK2, we undertook comprehensive co-crystal structure determination for the inhibitor set. Although some combinations of inhibitor and CDK did not yield well-diffracting crystals, a total of 11 structures could be solved and analyzed (Table S3).

Alvocidib, CGP74514a, and AZD5438 were co-crystallized with CDK1-cyclin B-Cks2 (Table S3). Despite repeated co-crystallization attempts we were unable to detect Dinaciclib and SU9516 in the CDK1 active site. As expected, the chromone, purine, and pyrimidine cores of Alvocidib (Figure 3A), CGP74514a (Figure 3B), and AZD5438 (Figure 3C), respectively, all form two hydrogen bonds with the CDK1 hinge through hydrogen bonds to the main-chain amide of L83 and carbonyl of E81 while being held in a hydrophobic sandwich between L135 and A31. The cyclohexamine moiety of CGP74514a occupies the ribose binding pocket of CDK1, forming an additional hydrogen bond with the main-chain carbonyl of Q132 (Figure 3B). Alvocidib sits further back in the CDK1 active site than CGP74514a (compare panels Figures 3A and 3B) and forms a network of interactions through the piperidinol moiety with K33 and D146 within the phosphate binding pocket. The alkylated imidazole group of AZD5438 is also accommodated within the phosphate binding pocket and is stabilized through hydrophobic interactions with V18 (Figure 3C). All three inhibitors form hydrophobic interactions with the CDK1 gatekeeper residue F80 that forms the back corner of the active site cavity. The ethyl moiety of CGP74514a stacks against the phenyl ring, while the pyrimidine and chromone cores of AZD5438 and Alvocidib, respectively, make ring edge-face interactions.

Toward the solvent-exposed selectivity surface on the C-terminal CDK lobe, the phenylmethylsulfone group of AZD5438 forms a network of interactions (Figure 3C). A hydrogen bond is made through the main-chain NH of D86 and hydrophobic interactions with the side chain of I10 of the P loop act as a brace between the two lobes. Both Alvocidib (Figure 3A) and CGP74514a (Figure 3B) harbor a chlorophenyl group in this region; however, they adopt different poses. Alvocidib sits higher in the pocket, which allows the chlorophenyl ring to rotate toward the inhibitor core to take advantage of interactions with V18 and I10 of the P loop. In contrast, the CGP74514a chlorophenyl group is rotated away from the core, and, as it cannot be accommodated intra-molecularly, sits up against the main chain of M85 beyond the hinge region.

Superposition of the CDK1-cyclinB-Cks2 structures shows that inhibitor binding drives the P loop to adopt a number of

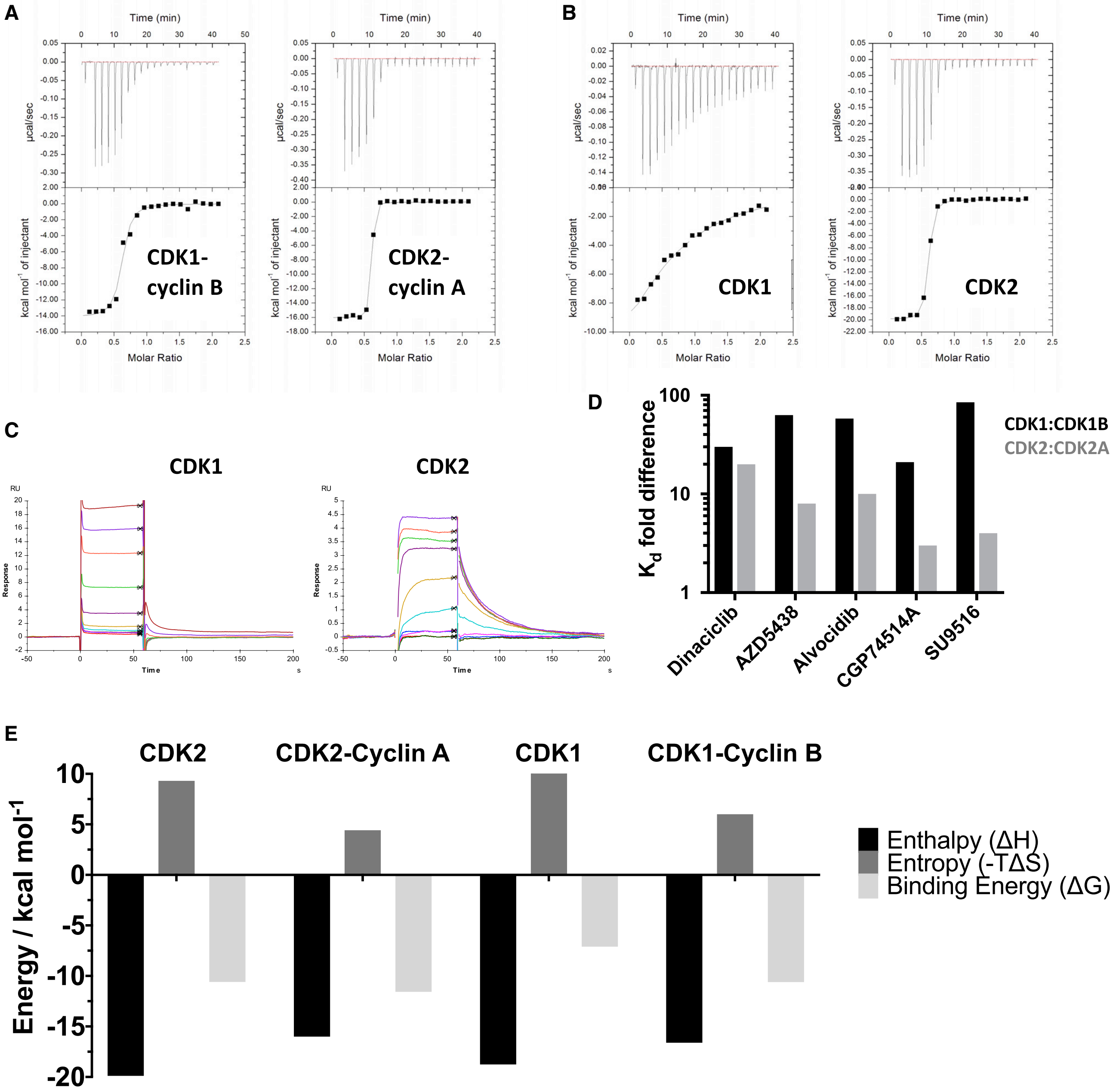

Figure 2. Inhibitor Binding to CDK1 and CDK2

(A and B) Isothermal titration calorimetry (ITC) thermograms to assess AZD5438 binding to CDK1-cyclin B and CDK2-cyclin A (A) and cyclin-free CDK1 and CDK2 (B). For each sample, CDK1 and CDK2 were phosphorylated (on T161 or T160, respectively). AZD5438 shows reduced binding to cyclin-free CDK1 compared with CDK1-cyclin (B).

(C) Surface plasmon resonance (SPR) studies to determine the binding of AZD5438 to CDK1 and CDK2. Unphosphorylated CDK1 and CDK2 as GST fusions were immobilized on the SPR chip via anti-GST antibody coupling. Accompanying sets of ITC thermograms and SPR traces that evaluate Dinaciclib, SU9516, Alvocidib, and CGP74514A binding are presented in Figure S2.

(D) Bar chart to compare the fold difference in binding affinity between cyclin-free CDK1 and CDK2 and their cyclin-associated forms. CDK1:CDK1-cyclin B and CDK2:CDK2-cyclin A ratios are shown in black and gray, respectively. ITC experiments conducted in the presence of Cks2 are presented in Figure S3.

(E) ITC-derived energetic experimental data (ΔH, -TΔS, and ΔG) for the binding of AZD5438 to CDK1 and CDK2 and their respective cognate cyclins.

alternative poses (Figure 3D), all of which are well defined in the electron density and none of which have refined to particularly high crystallographic temperature factors. A comparison of the P loop in the Alvocidib- and AZD5438-bound complexes shows a “tucked-in” Y15 accommodated within the active site, reminiscent of the apo CDK1-cyclin B-Cks2 structure. However, in the CGP74514a complex, Y15 does not fit and is forced to adopt a “popped-out” pose in which it coordinates to E163 on the

Table 2. Inhibitor Binding to CDK1 and CDK2

	SPR K_d (nM)		ΔT_m (°C)	
	CDK1	CDK2	CDK1	CDK2
Dinaciclib	1,810 ± 150	78 ± 16	0.5	12.6
AZD5438	1,560 ± 220	43 ± 8.1	−2.6	8.9
Alvocidib	1,080 ± 200	650 ± 230	0.9	7.3
CGP74514A	13,500 ± 1,000	312 ± 52	−3.3	5.2
SU9516	5,840 ± 720	56 ± 26	−1.5	11.7

Inhibitor binding was measured by surface plasmon resonance (SPR). The ΔT_m values were measured by differential scanning fluorimetry (DSF). ΔT_m represents the shift in T_m of monomeric CDK1 and CDK2 in the absence and presence of each inhibitor. SPR and DSF measurements were made with unphosphorylated and phosphorylated monomeric CDKs, respectively. SPR experiments were carried out in duplicate, and values are quoted ± SD.

activation loop. This rearrangement may penalize CGP74514a binding and contribute to its reduced potency toward CDK1 compared with Alvocidib and AZD5438.

A comparison of the binding modes of each of these three inhibitors to CDK1-cyclin B-Cks2 and to CDK2-cyclin A (Figure S5) reveals that each inhibitor exploits very similar interaction networks to bind to both complexes, consistent with their comparable binding affinities measured by ITC (Tables 1 and S2). A relative displacement of the C helix in the CDK1-cyclin B-Cks2 complexes compared with CDK2-cyclin A (Figure S5) is consistent with that reported previously (Martin et al., 2017; Brown et al., 2015), being far smaller than the displacement that accompanies cyclin binding and accommodated without affecting contacts made to the inhibitors described here.

As the differences in the inhibitor affinities for cyclin-free CDK1 and CDK2 are more profound, we next sought to rationalize these values by attempting to co-crystallize each inhibitor with cyclin-free CDK1. These structures, taken together with structures for the matching CDK2-inhibitor co-complexes, would allow a comparison of the molecular determinants that mediate inhibitor binding to cyclin-free CDK1 and CDK2.

Structures of Dinaciclib and AZD5438 Bound to Cyclin-free CDK1 Reveal Analogous Binding Modes to those Observed when Bound to Cyclin-free CDK2

Crystals of the CDK1-Cks2 complex were grown in the presence of each inhibitor. However, only two of the resolved structures contained unambiguous density for bound inhibitor (Figure 4; Table S3). CDK1-Cks2-Dinaciclib and CDK1-Cks2-AZD5438 crystallized in different lattices, which differ from the previously published apo CDK1-Cks1 lattice (Brown et al., 2015).

Dinaciclib binds to cyclin-free CDK1 through the core pyrazolo moiety, forming two hydrogen bonds with the main-chain NH and carbonyl of L83 (Figure 4A). The ethyl group pendant to the pyrazolo core is positioned up against F80, while the core is sandwiched between the two lobes through hydrophobic interactions with A31, V18, I10, V64, A145, and L135. The 2-hydroxyethyl group of the piperidine ring interacts with K33, which stabilizes D146 and potentially forms an intramolecular hydrogen bond with the pyrazolo core. Extending from the hinge toward the solvent-exposed selectivity surface, the Dinaciclib pyridine oxide group stacks up against the main chain of I10 through the face of the aromatic ring. Comparing the Dinaciclib-bound CDK1-Cks2 structure with that of apo CDK1-Cks2 we observe a slight, hydrophobicity-driven P loop movement toward the inhibitor, which results in a reduction of the active site volume, explaining the interactions we observe in the crystal structure. However, a global superposition of CDK1-Cks2-Dinaciclib and CDK2-Dinaciclib (PDB: 4KD1; Martin et al., 2013) (Figure 4B) reveals little disparity between the two active site structures (global superimposition root-mean-square deviation [RMSD] = 0.720 Å) and does not identify marked differences in interactions that would trivially explain the 20-fold difference in Dinaciclib potency toward CDK2 and CDK1.

AZD5438 binds ~170-fold more tightly to cyclin-free CDK2 than to CDK1 as determined by ITC measurements (Table 1). Through its pyrimidine core this inhibitor makes two NH and carbonyl L83 main-chain hydrogen bonds, while the aromatic core is held in place by interactions with CDK1 F80, L135, and A31 (Figure 4C). The phenylmethylsulfone moiety forms a potential hydrogen bond with K89 through the sulfone, while the phenyl ring sits up against I10 and is stabilized via an intramolecular interaction with the isopropyl group of the alkylated imidazole. These interactions are conserved in the cyclin-free CDK2-AZD5438 complex (Table S3), and, again, the two structures superimpose very well (global superposition RMSD = 1.043 Å).

We investigated the possibility that crystal contacts might be limiting the conformational responses of cyclin-free CDK1 and CDK2 to inhibitor binding—and thereby concealing a trivial explanation for the relatively low inhibitor binding affinity of cyclin-free CDK1—by comparing the available crystal forms of CDK1 and CDK2 (Betzi et al., 2011; Bourne et al., 1996; Brown et al., 2015; Jeffrey et al., 1995). Cyclin-free CDK2 crystallizes in one major form with space group $P2_12_12_1$. Although a significant fraction of the CDK2 surface area in this crystal form participates in crystal contacts, large conformational differences involving the glycine lid, the C helix, and the activation segment have been described for different CDK2-inhibitor complexes (for example, see Figure S6A), suggesting that this crystal form is capable of accommodating significantly different kinase conformations. Thus, it is reasonable to conclude that the conformation observed in our co-crystallization experiments with cyclin-free CDK2 are not unduly constrained by lattice contacts. Taking non-crystallographic symmetry into account, we have so far observed cyclin-free CDK1 subunits in five distinct lattice environments in two unrelated crystal forms. In each lattice environment, the conformation of the major regulatory structural elements is well preserved, and therefore unlikely to be driven by specific local crystal contacts (Figure S6B). Thus, while we cannot exclude an effect of the crystalline environment on the structures described here, we think such an effect is unlikely to dominate.

Comparing AZD5438 bound to CDK1-Cks2 (Figure 4C) and to CDK1-cyclin B-Cks2 (Figure 3C, overlaid in Figure 4D), it is difficult to rationalize the ~60-fold difference in affinity based purely on a comparison of the identified interactions. However, a plausible hypothesis is that cyclin binding reduces the overall entropy of CDK1 and pre-arranges the CDK1 active site to make optimal interactions with the ligand that results in greater affinity toward small-molecule inhibitors. This hypothesis was tested using a

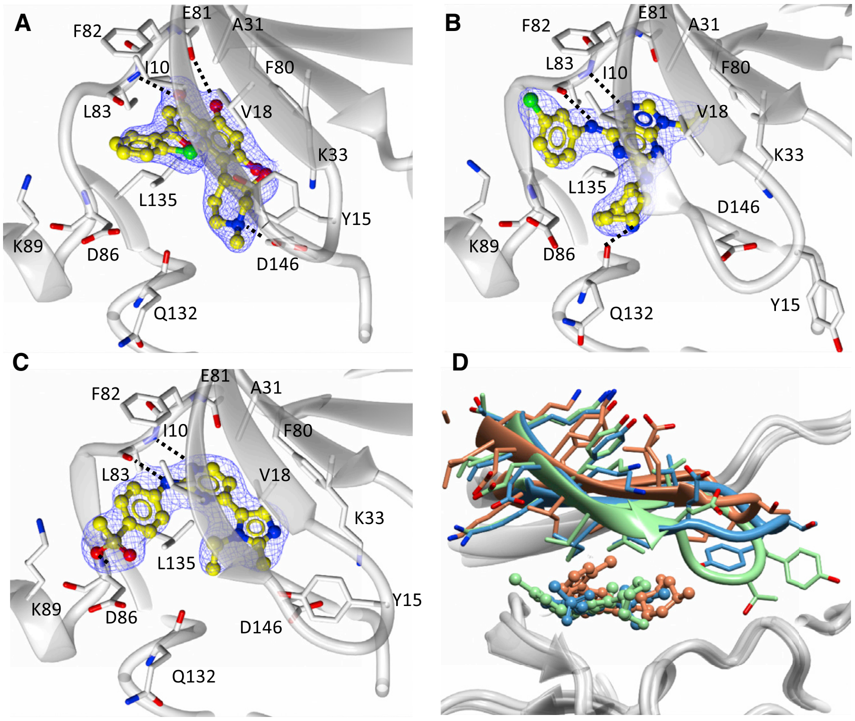

Figure 3. Crystal Structures of CDK1-Cyclin B-Cks2 Bound to Alvocidib, AZD5438, and CGP74514A

(A–C) (A) Alvocidib, (B) CGP74514A, and (C) AZD5438 all mimic the adenosine of ATP and bind to the hinge region of CDK1 to form a number of interactions within the binding pocket. The active site of CDK1 is shown in white ribbons with key residues depicted as cylinders. Inhibitors are shown in ball and stick mode (carbon atoms yellow and chlorine atoms green), with the supporting $2F_0$-Fc electron density maps rendered in blue mesh contoured at 1σ around the inhibitor. Potential hydrogen bonds are shown in black dotted lines.

(D) Global superimposition of the three inhibitor bound CDK1-cyclin B-Cks2 co-crystal structures to show the various P loop conformations adopted to accommodate inhibitor binding. CDK1 residues 11–17 in each inhibitor co-complex structure and the bound inhibitors are colored as follows: Alvocidib (pink), CGP74514A (lime green), and AZD5438 (blue).

more sophisticated approach for correlating the structural and biophysical results as described below.

Differences in the Stability and Flexibility of Cyclin-free CDK1 and CDK2 Contribute to the Observed Differences in ATP-Competitive Inhibitor Binding

The relatively minor differences between the crystal structures of cyclin-free CDK1 and CDK2 bound to AZD5438 and Dinaciclib are hard to reconcile with the large differences in affinity observed in our biophysical studies. One possible explanation is that the differences in affinity arise from dynamic properties of the different CDKs, and/or from structural differences between CDK1 and CDK2 complexes that are not expressed in the crystalline state due to constraints imposed by lattice contacts. If such properties underlie the biophysical results, then a system-wide molecular dynamics (MD) energetic calculation should be able to predict the affinities. Accordingly, we attempted affinity predictions using an MD-molecular mechanics energies combined with the Poisson-Boltzmann and surface area continuum solvation (MM/PBSA) approach (Kumari et al., 2014).

The relative predicted affinities for Dinaciclib and AZD5438 binding to cyclin-free CDK1 and CDK2 were consistent with our experimental findings, being substantially tighter for interactions made with CDK2 than those made with CDK1 (Figure S7). However, the "control" computational experiment of predicting AZD5438 affinity for CDK1-cyclin B and CDK2-cyclin A did not provide confidence in the significance of these calculations: the calculations suggested that CDK1-cyclin B should bind AZD5438 several orders of magnitude more tightly than does CDK2-cyclin A (Figure S7). Indeed, even when attempting to include a configurational entropy term in our analysis (Karplus and Kushick, 1981; Hou et al., 2011), we were unable to recapitulate the full set of experimental ITC observations. Therefore, we conclude that MM/PBSA calculations at the degree of sophistication applied here are not able to capture the complex nature of the comparative binding events involved. It may be that more computationally rigorous methods (e.g., potential of mean force/umbrella sampling; Steinbrecher and Labahn, 2010; Sun et al., 2015), that better sample the conformational energy landscape, and/or the energetic barriers between bound and unbound states, would enable a quantitative predictive understanding.

Interaction Networks that Might Impact Inhibitor Binding

Because our data suggested that plastic structural properties might explain the observed range of binding affinities for AZD5438, we surveyed the extent to which the different structures demonstrate proper assembly of two hydrophobic spines that link the N- and C-terminal lobes of the kinase fold. These two spines (the "regulatory" R-spine and the "catalytic" C-spine) reconfigure dynamically to regulate the activity of several protein kinases (Taylor and Kornev, 2011) (Figure 5A). Our structural data show that cyclin-bound CDK1 and CDK2 form the two conserved hydrophobic spines (Figure 5B). However, in cyclin-free CDK1 and CDK2 we observe misalignment of the R-spine, due to displacement of L55 from the C helix, which may result in reduced inter-lobe stability of the cyclin-free CDK forms.

In addition, cyclin-bound CDK1 and CDK2 form a series of interactions between their respective N- and C-lobes through atoms that derive from (1) the extended hinge region of the C-lobe, (2) the base of the β sheet that comprises β1-5, (3) the C helix (which is part of the N-lobe), and (4) the activation loop (which forms part of the C-lobe). In total, these elements form 133 and 145 inter-lobe contacts (<4.0 Å) for CDK1-cyclin B and CDK2-cyclin A, respectively. This network of interactions correlates with enhanced stability of the kinase fold and could, in turn, enhance the binding of small-molecule inhibitors.

Although cyclin-free CDK1 and CDK2 form a broadly similar set of inter-lobe interactions, they show a significantly smaller

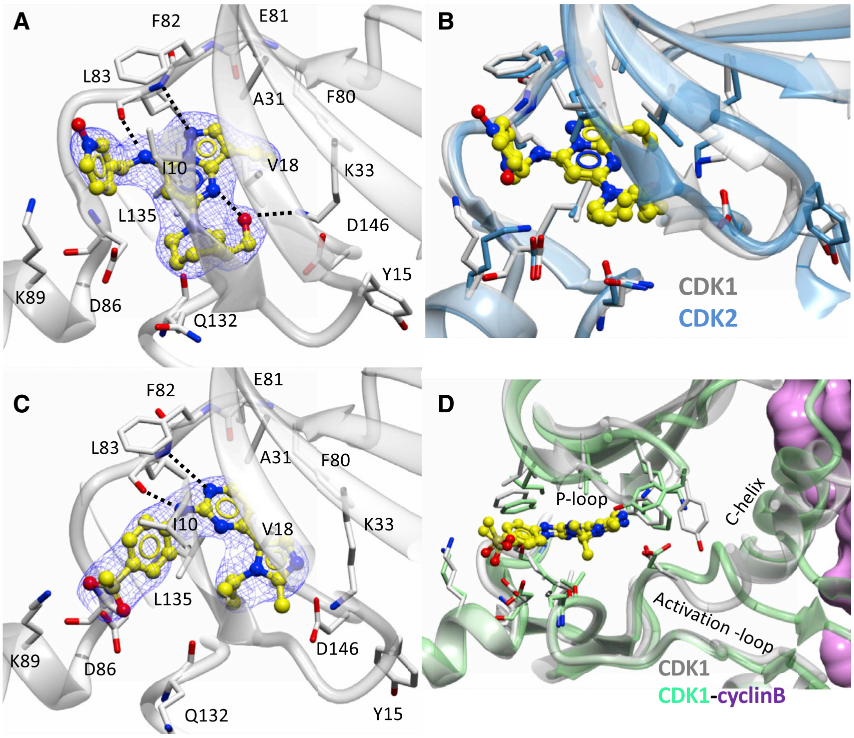

Figure 4. Characterization of Inhibitor Binding to CDK1

(A) Dinaciclib binds to the ATP binding pocket of CDK1-Cks2 through a series of hydrogen bonds and hydrophobic interactions that are comparable with those observed in the crystal structure of CDK2-Dinaciclib (PDB: 4KD1).

(B) The binding poses of Dinaciclib in the inactive CDK1-Cks2 and cyclin-free CDK2 ATP binding sites are conserved and anchored by the pyrimidine core.

(C) AZD5438 bound to cyclin-free CDK1-Cks2 through a series of hydrogen bonds and hydrophobic interactions.

The active sites of CDK1 and CDK2 are shown in white and blue ribbons, respectively, with key residues and inhibitors depicted in cylinder or yellow ball and stick styles, respectively. The $2F_0$-Fc electron density map is drawn as a blue mesh contoured at 1σ around the inhibitor. Potential hydrogen bonds are shown as black dotted lines.

(D) Comparison of the co-crystal structures of AZD5438 bound to cyclin-free and cyclin B bound active CDK1 to compare the key kinase motifs, namely the P loop, C helix, and activation segment. CDK1-cyclin B is drawn as a lime green ribbon and magenta surface.

number of contacts that involve the labile C helix and activation loop. Notably, however, residues T14 and Y15 from the P loop of cyclin-free CDK2 pack against the HRD motif of the CDK2 activation loop (Figure 5B) to form an interaction network that is not seen in cyclin-free CDK1. It may be that conformational stabilization that derives from these contacts contributes to the tighter binding of inhibitors to cyclin-free CDK2 than to cyclin-free CDK1. Overall, the number of inter-lobe contacts in cyclin-free CDK1 (104 contacts shorter than 4.0 Å) is the smallest of any state evaluated here, and compares with a count of 141 for cyclin-free CDK2.

Whether or not the specific contacts enumerated here contribute directly to enhanced inhibitor binding for CDK2 versus CDK1, they provide a structural basis for the suggestion that the conformational energy landscape of cyclin-free CDK1 differs from those of CDK2, CDK2-cyclin A, and CDK1-cyclin B in a way that might explain CDK1's relatively low propensity to bind small-molecule ligands.

DISCUSSION

The development of CDK inhibitors for clinical use is complicated by the challenge of achieving the best possible spectrum of activity against different CDK family members. In particular, a broad selectivity is thought to contribute to the dose-limiting toxicity that has led to the closure of a number of clinical programs (Asghar et al., 2015; Mariaule and Belmont, 2014; Whittaker et al., 2017). The recent successful CDK4/6 programs of IBRANCE, KISQALI, and VERZENIO are selective over CDK1 (Vijayaraghavan et al., 2017; Whittaker et al., 2017), and a comparison of the CDK4 and CDK1 active sites shows the potential for obtaining selectivity by exploiting sequence differences. In contrast, discriminating against CDK1 for a CDK2 drug discovery program appears more challenging because of the high degree of active site sequence identity between the two CDKs, and the similarity of their active site structures when bound to their cognate activating cyclins.

Our results suggest that targeting cyclin-free CDK2 could yield a window of selectivity over CDK1 that has not been accessible to drug discovery campaigns that target CDK2-cyclin complexes. The feasibility of generating an inhibitor that selectively binds to cyclin-free CDK2 has been suggested by results generated elsewhere (Alexander et al., 2015; Deng et al., 2014). These programs have developed compounds that bind tightly to cyclin-free CDK2 by exploiting an allosteric pocket and, in one case (Alexander et al., 2015), by altering the conformation of the DFG motif. Elsewhere, compounds that bind relatively weakly to monomeric CDK2 have revealed further space that can be exploited by inhibitors that bind outside the conventional ATP binding site (Betzi et al., 2011). However, no such inhibitor has yet been reported to provide potent inhibition of CDK2 kinase activity (compounds reported to be inactive at 10 μM).

Crystallographic structures reveal only minor differences in interactions between ATP-competitive inhibitors and cyclin-free and cyclin-bound forms of CDK1 and CDK2. From these differences, it is hard to explain the 100-fold difference in affinities of the inhibitors for the different protein targets. Accordingly, we have investigated whether the differences in affinity might be explained by the flexibility, plasticity, and entropic properties that can be interrogated in MD simulations of the different apo- and ligand-bound CDKs. These studies suggest that MD/MM-PBSA cannot provide a comprehensive predictive model for the ligand binding properties of the different CDKs in different states of cyclin binding. A qualitative indication of differing conformational energy landscapes for these targets was, however, indicated by an

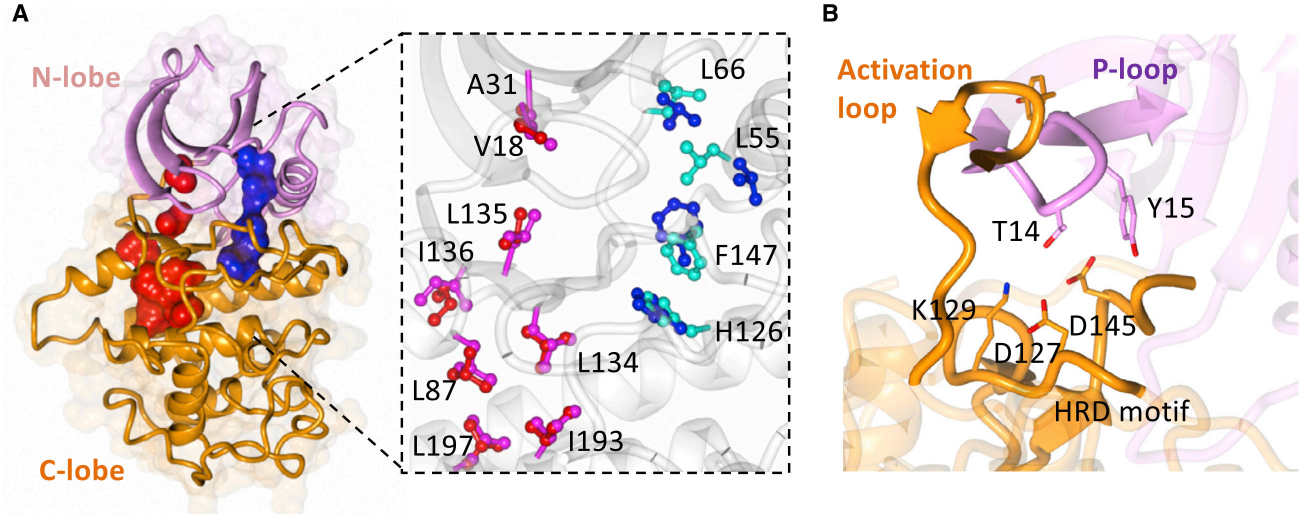

Figure 5. Plasticity of CDK Fold Regulated through the Hydrophobic Spine

(A) Inter-lobal stability of CDK fold, N-lobe (pink), C-lobe (orange), with hydrophobic regulatory spines of R-spine and C-spine shown in blue and red, respectively. Inset shows the C-spine and R-spine of CDK1 (red and blue) and CDK1-cyclinB (pink and cyan).

(B) Cyclin-free CDK2 stability through interactions of the P loop in the N-lobe (purple) and activation loop of the C-lobe (orange).

evaluation of their regulatory spine integrity (Taylor and Kornev, 2011) and an enumeration of their inter-lobe contacts. These analyses showed that contacts between the P loop and the activation segment of cyclin-free CDK2 might predispose it to inhibitor binding relative to cyclin-free CDK1, and that cyclin binding completes the formation of the regulatory spines of both kinases in a way that may contribute to the tighter binding of inhibitors to CDK-cyclin complexes. Overall, we conclude that the enhanced propensities of cyclin-free CDK2, cyclin-bound CDK2, and cyclin-bound CDK1 to bind to ligands, relative to that of cyclin-free CDK1, derives from a complex interplay between dynamic and plastic properties of cyclin-free CDK1.

A characteristic of protein kinases is their ability to signal the presence of ligands bound at the active site throughout the kinase domain to affect downstream kinase-dependent pathways (Kornev and Taylor, 2015). The ability to remodel the CDK active site underpins a variety of mechanisms that regulate CDK activity by phosphorylation (Morgan, 2007; Welburn et al., 2007) or binding to INK and CIP/KIP inhibitory proteins (Morgan, 2007; Pavletich, 1999). CDK4 retains an inactive conformation even when cyclin-bound and phosphorylated on the activation segment, suggesting that binding of the protein substrate is required in order for the Michaelis complex to form (Day et al., 2009). Taken together, these examples illustrate how sequence flexibility local to the active site and structural re-organization of the kinase domain are significant elements of CDK regulation. We hypothesize that, in addition to contributing to the unique activities of CDK1 as a cell-cycle regulator, the particular dynamic and plastic properties of CDK1 might also be exploited to successfully differentiate CDK1 from other CDKs in future cancer therapeutic design.

SIGNIFICANCE

This paper explores the adaptive nature of CDK1 and CDK1-cyclin B relative to CDK2 and CDK2-cyclin A by probing with a collection of ATP-competitive inhibitors. These inhibitors only modestly discriminate between cyclin-associated forms of CDK1 and CDK2, but bind much less tightly to cyclin-free CDK1 than to cyclin-free CDK2. These results hint at a different conformational energy landscape of cyclin-free CDK1 compared with cyclin-free CDK2, which is not apparent in cyclin-associated forms. This difference may underlie the essential cell-cycle role of CDK1, which can bind a sufficient set of cyclins to drive all phases of the cell cycle (Santamaria et al., 2007), and can phosphorylate suboptimal model substrates in a reconstituted system (Brown et al., 2015).

STAR★METHODS

Detailed methods are provided in the online version of this paper and include the following:

- KEY RESOURCES TABLE
- CONTACT FOR REAGENT AND RESOURCE SHARING
- METHOD DETAILS
  - Protein Expression and Purification
  - Crystallisation and Structure Determination
  - Isothermal Titration Calorimetry
  - Differential Scanning Calorimetry
  - Differential Scanning Fluorimetry
  - Kinase Activity Assays
  - Molecular Dynamics Simulations
  - Surface Plasmon Resonance
  - MM/PBSA Calculations
  - Entropy Calculations
- DATA AND SOFTWARE AVAILABILITY

SUPPLEMENTAL INFORMATION

Supplemental Information includes seven figures and three tables and can be found with this article online at https://doi.org/10.1016/j.chembiol.2018.10.015.

ACKNOWLEDGMENTS

We thank beamline staff at Diamond Light Source (Oxford), M. Ali for help in refinement of CDK2 structures, and A. Basle for assistance during X-ray data collection. We thank V. Frasca (Malvern Panalytical) for insightful discussion regarding DSC and Astex Pharmaceuticals for useful discussion regarding CDK inhibition. This research was supported by grants from Cancer Research UK (grant reference C2115/A21421), Newcastle Cancer Center and the JGW Patterson Foundation, and the Medical Research Council (grant reference MR/N009738/1).

AUTHOR CONTRIBUTIONS

M.P.M., J.A.E., and M.E.M.N. designed and supervised the project. S.K., D.J.W., L.-Z.W., and M.P.M. performed the protein production, ITC, SPR, differential scanning fluorimetry, and X-ray crystallography. N.J.T. performed the MD simulations and MM/PBSA calculations. All authors analyzed the data and D.J.W., N.J.T., M.P.M., J.A.E., and M.E.M.N. wrote the paper.

DECLARATION OF INTERESTS

The authors declare no competing financial interest. Some work in the authors' laboratory is supported by a research grant from Astex Pharmaceuticals.

Received: June 14, 2018
Revised: August 15, 2018
Accepted: October 11, 2018
Published: November 21, 2018

REFERENCES

Alexander, L.T., Mobitz, H., Drueckes, P., Savitsky, P., Fedorov, O., Elkins, J.M., Deane, C.M., Cowan-Jacob, S.W., and Knapp, S. (2015). Type II inhibitors targeting CDK2. ACS Chem. Biol. *10*, 2116–2125.

Asghar, U., Witkiewicz, A.K., Turner, N.C., and Knudsen, E.S. (2015). The history and future of targeting cyclin-dependent kinases in cancer therapy. Nat. Rev. Drug Discov. *14*, 130–146.

Au-Yeung, G., Lang, F., Azar, W.J., Mitchell, C., Jarman, K.E., Lackovic, K., Aziz, D., Cullinane, C., Pearson, R.B., Mileshkin, L., et al. (2017). Selective targeting of cyclin E1-amplified high-grade serous ovarian cancer by cyclin-dependent kinase 2 and AKT inhibition. Clin. Cancer Res. *23*, 1862–1874.

Baker, N.A., Sept, D., Joseph, S., Holst, M.J., and McCammon, J.A. (2001). Electrostatics of nanosystems: application to microtubules and the ribosome. Proc. Natl. Acad. Sci. U S A *98*, 10037–10041.

Berthet, C., Aleem, E., Coppola, V., Tessarollo, L., and Kaldis, P. (2003). Cdk2 knockout mice are viable. Curr. Biol. *13*, 1775–1785.

Betzi, S., Alam, R., Martin, M., Lubbers, D.J., Han, H., Jakkaraj, S.R., Georg, G.I., and Schonbrunn, E. (2011). Discovery of a potential allosteric ligand binding site in CDK2. ACS Chem. Biol. *6*, 492–501.

Bourne, Y., Watson, M.H., Hickey, M.J., Holmes, W., Rocque, W., Reed, S.I., and Tainer, J.A. (1996). Crystal structure and mutational analysis of the human CDK2 kinase complex with cell cycle-regulatory protein CksHs1. Cell *84*, 863–874.

Brown, N.R., Korolchuk, S., Martin, M.P., Stanley, W.A., Moukhametzianov, R., Noble, M.E.M., and Endicott, J.A. (2015). CDK1 structures reveal conserved and unique features of the essential cell cycle CDK. Nat. Commun. *6*, 6769.

Brown, N.R., Noble, M.E., Endicott, J.A., and Johnson, L.N. (1999). The structural basis for specificity of substrate and recruitment peptides for cyclin-dependent kinases. Nat. Cell Biol. *1*, 438–443.

Byth, K.F., Thomas, A., Hughes, G., Forder, C., McGregor, A., Geh, C., Oakes, S., Green, C., Walker, M., Newcombe, N., et al. (2009). AZD5438, a potent oral inhibitor of cyclin-dependent kinases 1, 2, and 9, leads to pharmacodynamic changes and potent antitumor effects in human tumor xenografts. Mol. Cancer Ther. *8*, 1856–1866.

Carlson, B.A., Dubay, M.M., Sausville, E.A., Brizuela, L., and Worland, P.J. (1996). Flavopiridol induces G1 arrest with inhibition of cyclin-dependent kinase (CDK) 2 and CDK4 in human breast carcinoma cells. Cancer Res. *56*, 2973–2978.

Coxon, C.R., Anscombe, E., Harnor, S.J., Martin, M.P., Carbain, B., Golding, B.T., Hardcastle, I.R., Harlow, L.K., Korolchuk, S., Matheson, C.J., et al. (2017). Cyclin-dependent kinase (CDK) inhibitors: structure-activity relationships and insights into the CDK-2 selectivity of 6-substituted 2-arylaminopurines. J. Med. Chem. *60*, 1746–1767.

Day, P.J., Cleasby, A., Tickle, I.J., O'Reilly, M., Coyle, J.E., Holding, F.P., McMenamin, R.L., Yon, J., Chopra, R., Lengauer, C., et al. (2009). Crystal structure of human CDK4 in complex with a D-type cyclin. Proc. Natl. Acad. Sci. U S A *106*, 4166–4170.

De Bondt, H.L., Rosenblatt, J., Jancarik, J., Jones, H.D., Morgan, D.O., and Kim, S.H. (1993). Crystal structure of cyclin-dependent kinase 2. Nature *363*, 595–602.

Deng, Y., Shipps, G.W., Jr., Zhao, L., Siddiqui, M.A., Popovici-Muller, J., Curran, P.J., Duca, J.S., Hruza, A.W., Fischmann, T.O., Madison, V.S., et al. (2014). Modulating the interaction between CDK2 and cyclin A with a quinoline-based inhibitor. Bioorg. Med. Chem. Lett. *24*, 199–203.

Desai, D., Wessling, H.C., Fisher, R.P., and Morgan, D.O. (1995). Effects of phosphorylation by CAK on cyclin binding by CDC2 and CDK2. Mol. Cell. Biol. *15*, 345–350.

Echalier, A., Endicott, J.A., and Noble, M.E. (2010). Recent developments in cyclin-dependent kinase biochemical and structural studies. Biochim. Biophys. Acta *1804*, 511–519.

Emsley, P., Lohkamp, B., Scott, W.G., and Cowtan, K. (2010). Features and development of Coot. Acta Crystallogr. D Biol. Crystallogr. *66*, 486–501.

Endicott, J.A., Noble, M.E., and Johnson, L.N. (2012). The structural basis for control of eukaryotic protein kinases. Annu. Rev. Biochem. *81*, 587–613.

Etemadmoghadam, D., Au-Yeung, G., Wall, M., Mitchell, C., Kansara, M., Loehrer, E., Batzios, C., George, J., Ftouni, S., Weir, B.A., et al. (2013a). Resistance to CDK2 inhibitors is associated with selection of polyploid cells in CCNE1-amplified ovarian cancer. Clin. Cancer Res. *19*, 5960–5971.

Etemadmoghadam, D., Weir, B.A., Au-Yeung, G., Alsop, K., Mitchell, G., George, J., Davis, S., D'Andrea, A.D., Simpson, K., Hahn, W.C., et al. (2013b). Synthetic lethality between CCNE1 amplification and loss of BRCA1. Proc. Natl. Acad. Sci. U S A *110*, 19489–19494.

Freemantle, S.J., and Dmitrovsky, E. (2010). Cyclin E transgenic mice: discovery tools for lung cancer biology, therapy, and prevention. Cancer Prev. Res. (Phila.) *3*, 1513–1518.

Hole, A.J., Baumli, S., Shao, H., Shi, S., Huang, S., Pepper, C., Fischer, P.M., Wang, S., Endicott, J.A., and Noble, M.E. (2013). Comparative structural and functional studies of 4-(thiazol-5-yl)-2-(phenylamino)pyrimidine-5-carbonitrile CDK9 inhibitors suggest the basis for isotype selectivity. J. Med. Chem. *56*, 660–670.

Homeyer, N., Horn, A.H., Lanig, H., and Sticht, H. (2006). AMBER force-field parameters for phosphorylated amino acids in different protonation states: phosphoserine, phosphothreonine, phosphotyrosine, and phosphohistidine. J. Mol. Model. *12*, 281–289.

Hornak, V., Abel, R., Okur, A., Strockbine, B., Roitberg, A., and Simmerling, C. (2006). Comparison of multiple Amber force fields and development of improved protein backbone parameters. Proteins *65*, 712–725.

Hou, T., Wang, J., Li, Y., and Wang, W. (2011). Assessing the performance of the MM/PBSA and MM/GBSA methods. 1. The accuracy of binding free energy calculations based on molecular dynamics simulations. J. Chem. Inf. Model. *51*, 69–82.

Imbach, P., Capraro, H.G., Furet, P., Mett, H., Meyer, T., and Zimmermann, J. (1999). 2,6,9-Trisubstituted purines: optimization towards highly potent and selective CDK1 inhibitors. Bioorg. Med. Chem. Lett. *9*, 91–96.

Jeffrey, P.D., Russo, A.A., Polyak, K., Gibbs, E., Hurwitz, J., Massague, J., and Pavletich, N.P. (1995). Mechanism of CDK activation revealed by the structure of a cyclinA-CDK2 complex. Nature *376*, 313–320.

Kabsch, W. (2010). XDS. Acta Crystallogr. D Biol. Crystallogr. *66*, 125–132.

CellPress

Kanska, J., Zakhour, M., Taylor-Harding, B., Karlan, B.Y., and Wiedemeyer, W.R. (2016). Cyclin E as a potential therapeutic target in high grade serous ovarian cancer. Gynecol. Oncol. *143*, 152–158.

Karakas, C., Biernacka, A., Bui, T., Sahin, A.A., Yi, M., Akli, S., Schafer, J., Alexander, A., Adjapong, O., Hunt, K.K., et al. (2016). Cytoplasmic cyclin E and phospho-cyclin-dependent kinase 2 are biomarkers of aggressive breast cancer. Am. J. Pathol. *186*, 1900–1912.

Karplus, M., and Kushick, J.N. (1981). Method for estimating the configurational entropy of macromolecules. Macromolecules *14*, 325–332.

Kim, S., Loo, A., Chopra, R., Caponigro, G., Huang, A., Vora, S., Parasuraman, S., Howard, S., Keen, N., Sellers, W., et al. (2013). Abstract PR02: LEE011: an orally bioavailable, selective small molecule inhibitor of CDK4/6– reactivating Rb in cancer. Mol. Cancer Ther. *12*, https://doi.org/10.1158/1535-7163.TARG-13-PR02.

Koff, A., Cross, F., Fisher, A., Schumacher, J., Leguellec, K., Philippe, M., and Roberts, J.M. (1991). Human cyclin E, a new cyclin that interacts with two members of the CDC2 gene family. Cell *66*, 1217–1228.

Kornev, A.P., and Taylor, S.S. (2015). Dynamics-driven allostery in protein kinases. Trends Biochem. Sci. *40*, 628–647.

Kroeger, P.T., Jr., and Drapkin, R. (2017). Pathogenesis and heterogeneity of ovarian cancer. Curr. Opin. Obstet.Gynecol. *29*, 26–34.

Kumari, R., Kumar, R., Open Source Drug Discovery, C., and Lynn, A. (2014). g_mmpbsa – a GROMACS tool for high-throughput MM-PBSA calculations. J. Chem. Inf. Model. *54*, 1951–1962.

Lane, M.E., Yu, B., Rice, A., Lipson, K.E., Liang, C., Sun, L., Tang, C., McMahon, G., Pestell, R.G., and Wadler, S. (2001). A novel cdk2-selective inhibitor, SU9516, induces apoptosis in colon carcinoma cells. Cancer Res. *61*, 6170–6177.

Lim, S., and Kaldis, P. (2013). Cdks, cyclins and CKIs: roles beyond cell cycle regulation. Development *140*, 3079–3093.

Lockwood, W.W., Stack, D., Morris, T., Grehan, D., O'Keane, C., Stewart, G.L., Cumiskey, J., Lam, W.L., Squire, J.A., Thomas, D.M., et al. (2011). Cyclin E1 is amplified and overexpressed in osteosarcoma. J. Mol. Diagn. *13*, 289–296.

Long, F., Nicholls, R.A., Emsley, P., Graaeulis, S., Merkys, A., Vaitkus, A., and Murshudov, G.N. (2017). AceDRG: a stereochemical description generator for ligands. Acta Crystallogr. D Struct. Biol. *73*, 112–122.

Malumbres, M. (2014). Cyclin-dependent kinases. Genome Biol. *15*, 122.

Mariaule, G., and Belmont, P. (2014). Cyclin-dependent kinase inhibitors as marketed anticancer drugs: where are we now? A short survey. Molecules *19*, 14366–14382.

Martin, M.P., Alam, R., Betzi, S., Ingles, D.J., Zhu, J.Y., and Schonbrunn, E. (2012). A novel approach to the discovery of small-molecule ligands of CDK2. Chembiochem *13*, 2128–2136.

Martin, M.P., Endicott, J.A., and Noble, M.E.M. (2017). Structure-based discovery of cyclin-dependent protein kinase inhibitors. Essays Biochem. *61*, 439–452.

Martin, M.P., Olesen, S.H., Georg, G.I., and Schonbrunn, E. (2013). Cyclin-dependent kinase inhibitor dinaciclib interacts with the acetyl-lysine recognition site of bromodomains. ACS Chem. Biol. *8*, 2360–2365.

McCoy, A.J., Grosse-Kunstleve, R.W., Adams, P.D., Winn, M.D., Storoni, L.C., and Read, R.J. (2007). Phaser crystallographic software. J. Appl. Crystallogr. *40*, 658–674.

McNicholas, S., Potterton, E., Wilson, K.S., and Noble, M.E. (2011). Presenting your structures: the CCP4mg molecular-graphics software. Acta Crystallogr. D Biol. Crystallogr. *67*, 386–394.

Merrick, K.A., Larochelle, S., Zhang, C., Allen, J.J., Shokat, K.M., and Fisher, R.P. (2008). Distinct activation pathways confer cyclin-binding specificity on Cdk1 and Cdk2 in human cells. Mol. Cell *32*, 662–672.

Morgan, D.O. (2007). The Cell Cycle Principles of Control (Primers in Biology) (New Science Press Ltd).

Murshudov, G.N., Skubak, P., Lebedev, A.A., Pannu, N.S., Steiner, R.A., Nicholls, R.A., Winn, M.D., Long, F., and Vagin, A.A. (2011). REFMAC5 for the refinement of macromolecular crystal structures. Acta Crystallogr. D Biol. Crystallogr. *67*, 355–367.

Parry, D., Guzi, T., Shanahan, F., Davis, N., Prabhavalkar, D., Wiswell, D., Seghezzi, W., Paruch, K., Dwyer, M.P., Doll, R., et al. (2010). Dinaciclib (SCH 727965), a novel and potent cyclin-dependent kinase inhibitor. Mol. Cancer Ther. *9*, 2344–2353.

Patch, A.M., Christie, E.L., Etemadmoghadam, D., Garsed, D.W., George, J., Fereday, S., Nones, K., Cowin, P., Alsop, K., Bailey, P.J., et al. (2015). Whole-genome characterization of chemoresistant ovarian cancer. Nature *521*, 489–494.

Pavletich, N.P. (1999). Mechanisms of cyclin-dependent kinase regulation: structures of Cdks, their cyclin activators, and Cip and INK4 inhibitors. J. Mol. Biol. *287*, 821–828.

Potterton, L., Agirre, J., Ballard, C., Cowtan, K., Dodson, E., Evans, P.R., Jenkins, H.T., Keegan, R., Krissinel, E., Stevenson, K., et al. (2018). CCP4i2: the new graphical user interface to the CCP4 program suite. Acta Crystallogr. D Struct. Biol. *74*, 68–84.

Russo, A.A., Jeffrey, P.D., and Pavletich, N.P. (1996). Structural basis of cyclin-dependent kinase activation by phosphorylation. Nat. Struct. Biol. *3*, 696–700.

Santamaria, D., Barriere, C., Cerqueira, A., Hunt, S., Tardy, C., Newton, K., Caceres, J.F., Dubus, P., Malumbres, M., and Barbacid, M. (2007). Cdk1 is sufficient to drive the mammalian cell cycle. Nature *448*, 811–815.

Shao, H., Shi, S., Huang, S., Hole, A.J., Abbas, A.Y., Baumli, S., Liu, X., Lam, F., Foley, D.W., Fischer, P.M., et al. (2013). Substituted 4-(thiazol-5-yl)-2-(phenylamino)pyrimidines are highly active CDK9 inhibitors: synthesis, X-ray crystal structures, structure-activity relationship, and anticancer activities. J. Med. Chem. *56*, 640–659.

Sousa da Silva, A.W., and Vranken, W.F. (2012). ACPYPE - AnteChamber PYthon parser interfacE. BMC Res. Notes *5*, 367.

Steinbrecher, T., and Labahn, A. (2010). Towards accurate free energy calculations in ligand protein-binding studies. Curr. Med. Chem. *17*, 767–785.

Sun, H., Tian, S., Zhou, S., Li, Y., Li, D., Xu, L., Shen, M., Pan, P., and Hou, T. (2015). Revealing the favorable dissociation pathway of type II kinase inhibitors via enhanced sampling simulations and two-end-state calculations. Sci. Rep. *5*, 8457.

Taylor, S.S., and Kornev, A.P. (2011). Protein kinases: evolution of dynamic regulatory proteins. Trends Biochem. Sci. *36*, 65–77.

Toogood, P.L., Harvey, P.J., Repine, J.T., Sheehan, D.J., VanderWel, S.N., Zhou, H., Keller, P.R., McNamara, D.J., Sherry, D., Zhu, T., et al. (2005). Discovery of a potent and selective inhibitor of cyclin-dependent kinase 4/6. J. Med. Chem. *48*, 2388–2406.

Van Der Spoel, D., Lindahl, E., Hess, B., Groenhof, G., Mark, A.E., and Berendsen, H.J. (2005). GROMACS: fast, flexible, and free. J. Comput. Chem. *26*, 1701–1718.

Vijayaraghavan, S., Moulder, S., Keyomarsi, K., and Layman, R.M. (2017). Inhibiting CDK in cancer therapy: current evidence and future directions. Target Oncol. *13*, 21–38.

Wang, J., Wang, W., Kollman, P.A., and Case, D.A. (2006). Automatic atom type and bond type perception in molecular mechanical calculations. J. Mol. Graph Model. *25*, 247–260.

Waterman, D.G., Winter, G., Parkhurst, J.M., Fuentes-Montero, L., Hattne, J., Brewster, A., Sauter, N.K., and Evans, G. (2013). The DIALS Framework for Integration Software. CCP4 Newsletter on Protein Crystallography (CCP4), pp. 16–19. http://cci.lbl.gov/publications/download/DIALS.pdf.

Welburn, J.P., Tucker, J.A., Johnson, T., Lindert, L., Morgan, M., Willis, A., Noble, M.E., and Endicott, J.A. (2007). How tyrosine 15 phosphorylation inhibits the activity of cyclin-dependent kinase 2-cyclin A. J. Biol. Chem. *282*, 3173–3181.

Whittaker, S.R., Mallinger, A., Workman, P., and Clarke, P.A. (2017). Inhibitors of cyclin-dependent kinases as cancer therapeutics. Pharmacol. Ther. *173*, 83–105.

Winn, M.D., Ballard, C.C., Cowtan, K.D., Dodson, E.J., Emsley, P., Evans, P.R., Keegan, R.M., Krissinel, E.B., Leslie, A.G., McCoy, A.J., et al. (2011). Overview of the CCP4 suite and current developments. Acta Crystallogr. D Biol. Crystallogr. *67*, 235–242.

STAR★METHODS

KEY RESOURCES TABLE

REAGENT or RESOURCE	SOURCE	IDENTIFIER
Chemicals, Peptides, and Recombinant Proteins		
Dinaciclib	Selleckchem	Cat. #S2768
AZD5438	Tocris	Cat. #3698
Alvocidib	Selleckchem	Cat. #S1230
CGP74514A	Calbiochem	Cat. #217696
SU9516	Selleckchem	Cat. #S7636
Critical Commercial Assays		
ADPglo™	Promega	Cat. #V6930
Deposited Data		
CDK1/Cks1	Brown et al., 2015	Protein databank (PDB) code: 4YC6
CDK1/cyclin B/Cks2 + AZD5438	This study	PDB: 6GU3
CDK1/cyclin B/Cks2 + Alvocidib	This study	PDB: 6GU2
CDK1/cyclin B/Cks2 + CGP74514A	This study	PDB: 6GU4
CDK1/Cks2 + Dinaciclib	This study	PDB: 6GU6
CDK1/Cks2 + AZD5438	This study	PDB: 6GU7
CDK2/cyclin A	Jeffrey et al., 1995	PDB: 1FIN
CDK2/cyclin A + Alvocidib	This study	PDB: 6GUB
CDK2/cyclin A + SU9516	This study	PDB: 6GUC
CDK2/cyclin A + AZD5438	This study	PDB: 6GUE
CDK2/cyclin A + CGP74514A	This study	PDB: 6GUF
CDK2/Cks1	Bourne et al., 1996	PDB: 1BUH
CDK2 + AZD5438	This study	PDB: 6GUH
CDK2 + CGP74514A	This study	PDB: 6GUK
CDK2	De Bondt et al., 1993	PDB: 1HCK
CDK2 + Dinaciclib	Martin et al., 2013	PDB: 4KD1
CDK2 + JWS648 + ANS	Betzi et al., 2011	PDB: 3PXZ
Recombinant DNA		
Plasmid: GST-CDK1	Brown et al., 2015	N/a
Plasmid: GST-CDK2 with CAK1p	Brown et al., 1999	N/a
Plasmid: GST-CDK2	Martin et al., 2012	N/a
Plasmid: Cyclin A (Human)	Brown et al., 2015	N/a
Plasmid: Cyclin A (Bovine)	Brown et al., 2015	N/a
Plasmid: Cyclin B	Brown et al., 2015	N/a
Plasmid: Cks2	Brown et al., 2015	N/a
Software and Algorithms		
AceDRG	Long et al., 2017	http://www.ccp4.ac.uk/dist/checkout/acedrg/doc/Manual.pdf
ACPYPE	Sousa da Silva and Vranken, 2012	https://github.com/llazzaro/acpype
AMBER	Homeyer et al., 2006	http://ambermd.org/
Antechamber	Wang et al., 2006	http://ambermd.org/
APBS	Baker et al., 2001	https://apbs-pdb2pqr.readthedocs.io/en/latest/downloads.html
Biacore s200 Evaluation Software	GE Healthcare	Hardware Cat. #29136649, software as sold currently Cat. #29310602
CCP4i2	Potterton et al., 2018; Winn et al., 2011	http://www.ccp4.ac.uk/

(*Continued on next page*)

Continued

REAGENT or RESOURCE	SOURCE	IDENTIFIER
CCP4MG	McNicholas et al., 2011	http://www.ccp4.ac.uk/MG/
Coot	Emsley et al., 2010	https://www2.mrc-lmb.cam.ac.uk/personal/pemsley/coot/
DIALS	Waterman et al., 2013	https://dials.github.io/
Graphpad Prism 6	Graphpad Software	https://www.graphpad.com/scientific-software/prism/
g_mmpbsa	Kumari et al., 2014	https://rashmikumari.github.io/g_mmpbsa/
GROMACS	Van Der Spoel et al., 2005	http://www.gromacs.org/
Origin 7.0	Origin and Malvern Panalytical	https://www.malvernpanalytical.com
Phaser	McCoy et al., 2007	http://www.ccp4.ac.uk/html/phaser.html
Protein Thermal Shift Software 1.3	Thermo Fisher Scientific	Cat. #4466037
Refmac5	Murshudov et al., 2011	http://www.ccp4.ac.uk/html/refmac5.html
XDS	Kabsch, 2010	http://xds.mpimf-heidelberg.mpg.de/

CONTACT FOR REAGENT AND RESOURCE SHARING

Further information and requests for resources and reagents should be directed to and will be fulfilled by the Lead Contact, Martin E. M. Noble (martin.noble@newcastle.ac.uk).

METHOD DETAILS

Protein Expression and Purification

Human CDK1 and CDK2, and Cks2, and human cyclin A and B sequences were expressed, purified and assembled as required into complexes as described (Brown et al., 2015). Full-length human CDK1 was expressed in insect cells either untagged (from pVL1393) or as a 3C-protease cleavable GST fusion. To prepare the GST fusion, the CDK1 sequence was initially cloned into pGEX6P-1 as a Sma1-EcoR1 fragment. A cassette to express the fusion protein was extracted from recombinant pGEX6P-1 and cloned into the transfer vector pVL1393 and verified by DNA sequencing. Recombinant virus was generated using Sf9 cells co-transfected with pVL1393GST-CDK1 and FlashBacGold DNA (Oxford Expression Technologies) mixed with transfection reagent (GeneJuice, Novagen) and grown in SF900 II SFM media (LifeTechnologies). CDK1 expression was subsequently optimized by screening different virus stock:cell ratios. To purify GST-CDK1, cell pellets were resuspended in mTBS buffer (50 mM Tris-HCl, 150 mM NaCl, 1 mM dithiothreitol (DTT), pH7.5) supplemented with 200 μM RNAase A, 200 μM DNAaseI and 200 μM $MgCl_2$, lysed by sonication then clarified by centrifugation (60,000 × g, 1 h). GST-CDK1 was subsequently purified by sequential affinity chromatography (glutathione-sepharose 4B, GE Healthcare), GST-3C cleavage (1:50 w/w at +4 °C overnight) and a final size-exclusion chromatography (SEC) step (Superdex 75 26/60 column, GEHealthcare equilibrated in mTBS). Preparation of human cyclin B1 residues 165–433 carrying the C167S/C238S/C350S mutations, human cyclin A2 and bovine cyclin A2 (residues 172-430 (differing in 8 residues from human) used in only ITC experiments because human cyclin A is unstable in the absence of CDK2) were expressed in recombinant *E. coli* cells and purified as described in Brown et al., 2015. Human Cks1 and Cks2 were expressed as His6-or GST-tagged proteins, respectively, and purified by sequential affinity (Ni-NTA or glutathione-sepharose 4B), 3C cleavage (in the case of the GST fusion) and SEC steps. To prepare the CDK1–Cks1 complex, purified His-tagged Cks1 was immobilized on Ni-NTA and used to capture untagged CDK1 from insect cell lysate. The complex was eluted with imidazole and further purified by SEC on a HR26/60 SD75 column (GE Healthcare) equilibrated in HBS (10 mM HEPES pH 7.5/150 mM NaCl/0.01% monothioglycerol (MTG)/0.01% sodium azide). To prepare the ternary CDK1–cyclin B-Cks2 complex, components were individually purified and then mixed in a molar ratio of 1:1.5:2, CDK1:cyclin B:Cks2. The final step to assemble the complex was carried out on a Superdex 75 HR26/60 size-exclusion column equilibrated in a modified Tris-buffered saline containing 1.0 M NaCl, 50 mM Tris-HCl, pH 8.0, 1 mM DTT. Fractions containing the desired complexes were pooled, and both were concentrated to circa 10–12 mg ml^{-1} by ultrafiltration. Both the CDK1–Cks1 and CDK1–cyclin B–Cks2 complexes can be successfully crystallized from frozen samples. Proteins were concentrated to circa 10–12 mg ml^{-1} and then fast frozen in aliquots in liquid nitrogen before storage at −80°C. CDK2 suitable for crystallography was prepared as described in (Martin et al., 2012). Human CDK2 (encompassing full length residues 1-298) was cloned into a pGEX6P1 vector (GE Healthcare) and then transformed and expressed in BL21(DE3)STAR *E. coli*, then grown at 37°C until an optical density of 0.4-0.6 before decreasing the temperature to 18°C with 0.2 mM IPTG (Isopropyl β-D-1-thiogalactopyranoside; Sigma) induction and incubation for a further 16-20 hours. Cells were then harvested by centrifugation (4,000 xg, 30 mins, 4°C) before resuspension in 50 mM HEPES, 150 mM NaCl, 2 mM DTT, pH 7.4. Cells suspensions were then supplemented with 2 ug/mL DNase I, 10 ug/mL RNase A, 25 ug/mL lysozyme and 5 mM $MgCl_2$ for to aid lysis. Cell suspensions were then lysed via sonication (5 mins total, pulsed 20s on, 40s off) and lysate clarified via centrifugation (40, 000 xg, 60 mins, 4°C). Recovered lysate was then bound to glutathione

sepharose 4B resin (GE Healthcare; pre-equilibrated in 50 mM HEPES, 150 mM NaCl, 2 mM DTT, pH7.4) via batch binding before elution in 50 mM HEPES, 150 mM NaCl, 2 mM DTT, 10 mM reduced glutathione, pH 7.4. Eluted GST-CDK2 proteins were then cleaved overnight (16 hours) at 4°C using 1:100 mass:mass of 3C protease:GST-CDK2. Cleaved product then underwent gel filtration (s75 preparative grade Superdex® HiLoad® (GE Healthcare)) with peak, GST-free fractions then buffer exchanged, using a HiTrap desalting column (GE Healthcare), into 100 mM 4:1 K_2:Na phosphate, 2 mM DTT, buffer and concentrated to 10 mg mL^{-1}.

Crystallisation and Structure Determination

Crystals of CDK1–cyclin B-Cks2 bound to -Alvocidib, -CGP74514a and -AZD5438 were grown and cryo-protected as described (Brown et al., 2015). Briefly, crystals were grown by adding each inhibitor in two-fold molar excess (which was also a >10-fold higher concentration than the inhibitor IC_{50}) to a low concentration solution of CDK1–cyclin B-Cks2 and then concentrating the sample to 10–12 mg ml^{-1}. Monomeric CDK1-Cks2-Dinaciclib and -AZD5438 complexes were prepared by concentrating CDK1-Cks2 and inhibitor stock solutions as described above. Subsequent crystallisation and cryo-protection was as described for the apo-CDK1-Cks1 complex (Brown et al., 2015). CDK2-cyclin A- Alvocidib, -CGP74514a, SU9516 and -AZD5438 crystals were grown and harvested as described (Coxon et al., 2017). Monomeric CDK2 crystals grown as described (Martin et al., 2012), were soaked with both AZD5438 and CGP74514A at 1mM, 10% DMSO for 2 days. The crystals were then harvested into a cryoprotectant of 30% ethylene glycol diluted from a 100% stock with precipitant solution. X-ray diffraction data were recorded at Diamond Light Source, Oxford, UK. The programs within the CCP4i2 software package (Potterton et al., 2018; Winn et al., 2011) were employed throughout the structure determination process. Data were processed using DIALS (Waterman et al., 2013) and XDS (Kabsch, 2010) within Xia2, while PHASER (McCoy et al., 2007) was employed for phasing and REFMAC (Murshudov et al., 2011) used during refinement. Model building was performed using COOT (Emsley et al., 2010) with AceDRG used for generation of inhibitor descriptions (Long et al., 2017). Figures were prepared using CCP4MG (McNicholas et al., 2011).

Isothermal Titration Calorimetry

Individual protein samples were exchanged into ITC buffer (40 mM HEPES, 500 mM NaCl, 0.25 mM TCEP, pH 8.0) at 4°C using a Hitrap desalting column (GE Healthcare Life Sciences, Chicago, IL, USA) (i.e. CDK1 and cyclin B were not combined to form a complex prior to buffer exchange). Protein concentration was then determined at A_{280} nm using a Nanodrop 2000 (Thermoscientific, Waltham, MA, USA) and sequence derived extinction coefficients (http://web.expasy.org/protparam/). Proteins and compounds were degassed at 30°C directly before analysis. Compounds at 100 μM prepared in ITC buffer (40 mM HEPES, 500 mM NaCl, 0.25 mM TCEP, pH 8.0) and to a final DMSO concentration of 1% were titrated into 200 μL protein solutions prepared at 10 μM, 1% DMSO in the cell. All experiments used phosphorylated forms of CDK1 and CDK2 (on T161 or T160 respectively). For experiments carried out in the absence of cyclin, proteins were prepared on ice to a final concentration of 10 μM in ITC buffer and supplemented with 1% DMSO. Regarding CDK-cyclin or CDK-Cks2 titrations, complexes were prepared from individual buffer exchanged components at 1:1 equimolar concentrations (10 μM each, accounting for 1:1 stoichiometry) and incubated on ice for 30 mins prior to degassing and analysis. All ITC measurements were carried out at 30°C on a Microcal ITC 200 (Malvern Instruments Ltd, Malvern, UK). Titrations began with an initial 0.5 μL injection followed by 19 x 2 μL injections of compound, with 120 s spacing between each injection, and stirred at 1000 rpm throughout the experiment. Data were then analysed using Origin 7.0 (OriginLab Corp., Burlington, NC, USA) and fit with the one-set-of-sites model to calculate the dissociation constant and the error of the fit. Data are representative of at least two separate experiments from two separate biological preparations of protein.

Differential Scanning Calorimetry

The stability of the monomeric phosphorylated CDK1 and CDK2 were assessed by differential scanning calorimetry (DSC) using the Microcal VP-DSC (Malvern Instruments Ltd, Malvern, UK). Proteins were desalted in DSC buffer (50 mM HEPES, 150 mM NaCl, 0.25 mM TCEP, pH 7.4) at 4°C using a Hitrap desalting column (GE Healthcare Life Sciences, Chicago, IL, USA). Protein concentration was then determined at A_{280} nm using a Nanodrop 2000 (Thermoscientific, Waltham, MA, USA) and sequence derived extinction coefficients (http://web.expasy.org/protparam/). Prior to analysis, buffer equilibration was carried out overnight to obtain a stable baseline. Six overnight buffer equilibration scans were carried out using a starting temperature of 25°C, and an end temperature of 90°C at a rate of 90°C/hour. The pre-scan thermostat was set to 15 and the post-scan thermostat set to 0. Using the same temperature start- and end-points and rate, protein stability testing was carried out using 700 μL of CDK1 or CDK2 at 23 μM. Buffer-buffer equilibration runs were subtracted from protein-buffer scans, normalised for protein concentration and fit to a non-two state model using Origin 7.0 (OriginLab Corp., Burlington, NC, USA) software to calculate the melting temperature (T_m) and corresponding error of the fit. Experiments were conducted in singletons.

Differential Scanning Fluorimetry

The stability of the CDK1 and CDK2 inhibitor complexes were assessed by differential scanning fluorimetry (DSF) using a ViiA7 Real-Time PCR system (Applied Biosystems). Purified CDK1 (4 μM) and CDK2 (6 uM) in their respective gel filtration buffers were assayed in the presence of 10 μM inhibitor in 8% DMSO, in triplicate, in a 384-well plate. SyPRO Orange (x10) (Invitrogen) was used as the fluorescent probe, and fluorescence was measured using the ROX reporter channel (excitation λ=470 nm, emission λ = 586nm). Complex stability was assessed by increasing the temperature from 25 to 95°C through 1°C increments. The resulting data was plotted and the inflection point (Tm) calculated using the Boltzmann equation in Applied Biosystems software.

Kinase Activity Assays

Evaluation of the inhibitors were carried out in ADP-Glo^{TM} format essentially as described previously (Brown et al., 2015). Briefly, the 5 inhibitors in the study were titrated against phosphorylated CDK2–cyclin A and CDK1–cyclin B, with reaction initiated through addition of ATP and substrate peptide HHASPRK (Enzo Scientific). ATP to ADP conversion was assessed using the ADP-Glo assay (Promega) essentially as described by the manufacturers. In brief, reactions were carried out at room temperature in 40 mM Tris-HCl pH 7.5, containing 20 mM $MgCl_2$, 0.1 mg ml^{-1} BSA, using 4 nM CDK1-cyclinB or 1.5 nM CDK2–cyclin A incubated with inhibitors through a 12 points (0-6 μM) titration. Reactions were initiated by the addition of 20 μM ATP and 20 μM peptide. All activity assays were performed in triplicate in white low volume 384-well plates using a PheraStar plate reader (BMG). IC_{50} values were obtained by fitting the data to the equation Y=1/(1+10^((LogIC_{50}-[inhibitor])*N)) using PRISM (GraphPad).

Molecular Dynamics Simulations

The complexes of CDK1-AZD5438 and -Dinaciclib was derived by removing Cks2 and all water and solvent molecules. Complexes of CDK2-AZD5438 and CDK2-Dinaciclib (PDB: 4KD1), and complexes for CDK1-Cyclin B-AZD5438 and CDK2-Cyclin A-AZD5438 were prepared by removal of water and any additional solvent molecules. Parameters for phosphothreonine in complexes CDK2-AZD5438 and CDK2-CyclinA-AZD5438 were derived from Homeyer et al.'s published set for AMBER (Homeyer et al., 2006). Ligand topologies were prepared in Antechamber (Wang et al., 2006) from AmberTools16 and converted to GROMACS format using ACPYPE (Sousa da Silva and Vranken, 2012). Simulations were performed in GROMACS 5.1.4 (Van Der Spoel et al., 2005) using 1 NVIDIA GeForce GTX 1008 GPU and 10 Intel Xenon E5-2630 CPU threads. Each system was parameterised in the AMBER99SB (Hornak et al., 2006) forcefield and solvated in a cubic box with a 10 Å shell of TIP3P water. The systems were then neutralised using NaCl to a final concentration of 0.5 M to correspond to the biophysical experimental conditions, followed by steepest descent energy minimisation to a target Fmax of < 1000 kJ mol^{-1} nm^{-1}, achieved in under 2000 steps in all cases. Each system was subject to 200 ps equilibration at 300 K in the NVT ensemble using 2 fs steps. Equilibration was continued in the NPT ensemble for 500 ps using 2 fs steps. Atom positions were restrained during equilibration and released for production MD. Three production MD runs were performed using the equilibrated system as a starting point for a total of 10 ns in 2 fs steps utilising Nose-Hoover temperature coupling with a Parinello-Rahman pressure coupling. Energies and co-ordinates were written every 5000 steps (10 ps) for analysis. Convergence was determined by tracking the LJ and coulombic energies throughout the simulation, as well as the RMSD of both the protein alpha-carbons and the drug itself and the best converged run(s) were utilised for MM/PBSA calculation.

Surface Plasmon Resonance

SPR ligand interactions assays were performed on a Biacore S200 (GE Healthcare Life Sciences) at 20°C using multi-cycle settings. GST-tagged unphosphorylated CDK1 and CDK2 were immobilised onto the surface of a CM5 chip pre-immobilised with anti-GST antibody, provided as part of the GST-capture kit (GE Healthcare Life Sciences, catalogue number BR100223) following the GST immobilisation kit instructions. As a control, recombinant GST was immobilised onto the designated control flow cell. GST, GST-CDK1 and GST-CDK2 were then prepared at concentrations of 20 μg/mL, 50 μg/mL and 50 μg/mL respectively in DMSO-free SPR running buffer (20 mM HEPES, 150 mM NaCl, 0.01% Tween®-20, pH 7.4) and injected onto separate flow cells eliciting response units (RUs) of 620, 1900 and 2244 RUs, respectively. Preceding injection of each compound, three cycles of buffer injections were carried out over all surfaces (30 μL/min for 60 s with 360 s dissociation). Compounds were then injected over all surfaces (from low to high concentration) for 60 s at 30 μL/min before being allowed to dissociate for 360 s to record dose-responses. 11 (GST-CDK2) or 12 (GST-CDK1) concentrations (0 nM, 0.05 nM, 0.19 nM, 0.76 nM, 3.05 nM, 12.21 nM, 48.83 nM, 195.31 nM, 781.25 nM, 3125 nM and 12500 nM, with an extra concentration of 50000 nM for GST-CDK1) of compound were prepared at 1% DMSO in 20 mM HEPES, 150 mM NaCl, 0.01% Tween®-20, pH 7.4 in series across all flow cells using solvent correction. The experiment was conducted in SPR running buffer (20 mM HEPES, 150 mM NaCl, 0.01% Tween-20®, 1% DMSO, pH 7.4) and used an 8-point DMSO solvent correction (preparation of DMSO in DMSO-free SPR buffer to final concentrations of: 0.2%, 0.4%, 0.6%, 0.8% 1%, 1.2%, 1.4%, 1.6%) to account for any bulk flow interactions. Similarly, all responses were subtracted from the GST immobilised reference flow cell. Responses were analysed using Biacore s200 Evaluation Software (GE Healthcare Life Sciences, Chicago, IL, USA) using affinity fit to determine the K_d and standard error (SE) of the fit. Data are representative of two technical replicates.

MM/PBSA Calculations

The g_mmpbsa tool (Kumari et al., 2014) was utilised to calculate the binding energy between protein and ligand via APBS v. 1.4.1(Baker et al., 2001) using a solute dielectric constant of 1 and a solvent dielectric constant of 80. The final 4 ns of converged simulation time was used for binding energy determination. Parameters such as temperature and ion concentration were matched to the simulation parameters. Output energies were converted from kJ mol^{-1} to kcal mol^{-1}, and log_{10} fold-changes calculated using $\Delta\Delta G$/1.4 kcal mol^{-1}.

Entropy Calculations

The configurational entropy of each system was calculated as the quasiharmonic approximation of the entropy(Karplus and Kushick, 1981), as calculated in GROMACS based on the method of Karplus and Kushik. A covariance matrix was generated using gmx covar for each production MD run, based on the alpha-carbons of the protein component of each system, which was subsequently used to calculate the entropy using gmx anaeig with a specified temperature of 300K. The entropy for each complex was then averaged and

used to calculate an estimated enthalpy, with the MM/PBSA binding energy substituted for ΔG and the configurational entropy for ΔS.

DATA AND SOFTWARE AVAILABILITY

All crystallographic results were deposited to and are accessible from the Protein Data Bank (PDB) https://www.rcsb.org/.

PDB accession codes for all structures derived from this study are as follows:

CDK1/cyclin B/Cks2 + AZD5438 (PDB: 6GU3), CDK1/cyclin B/Cks2 + Alvocidib (PDB: 6GU2), CDK1/cyclin B/Cks2 + CGP74514A (PDB: 6GU4), CDK1/Cks2 + Dinaciclib (PDB: 6GU6), CDK1/Cks2 + AZD5438 (PDB: 6GU7), CDK2/cyclin A + Alvocidib (PDB: 6GUB), CDK2/cyclin A + SU9516 (PDB: 6GUC), CDK2/cyclin A + AZD5438 (PDB: 6GUE), CDK2/cyclin A + CGP74514A (PDB: 6GUF), CDK2 + AZD5438 (PDB: 6GUH), CDK2 + CGP74514A (PDB: 6GUK).

PDB accession codes for structures used within, but not derived from, this study:

CDK1/Cks1 (PDB: 4YC6), CDK2/cyclin A (PDB: 1FIN), CDK2/Cks1 (PDB: 1BUH), CDK2 (PDB: 1HCK), CDK2 + Dinaciclib (PDB: 4KD1), CDK2 + JWS648 + ANS (PDB: 3PXZ).

Cell Chemical Biology, Volume *26*

Supplemental Information

Differences in the Conformational Energy Landscape of CDK1 and CDK2 Suggest a Mechanism for Achieving Selective CDK Inhibition

Daniel J. Wood, Svitlana Korolchuk, Natalie J. Tatum, Lan-Zhen Wang, Jane A. Endicott, Martin E.M. Noble, and Mathew P. Martin

Supplementary Information for

Differences in the conformational energy landscape of CDK1 and CDK2 suggest a mechanism for achieving selective CDK inhibition

Daniel J. Wood, Svitlana Korolchuk, Natalie J. Tatum, Lan-Zhen Wang, Jane A. Endicott, Martin E.M. Noble and Mathew P. Martin

Lead contact: Martin E.M. Noble
Email: martin.noble@ncl.ac.uk

This PDF file includes:

Supplementary text
Figs. S1 to S7
Tables S1 to S3

Other supplementary materials for this manuscript include the following:

Table S3. Summary of data collection and structure refinement, related to Figures 3-5

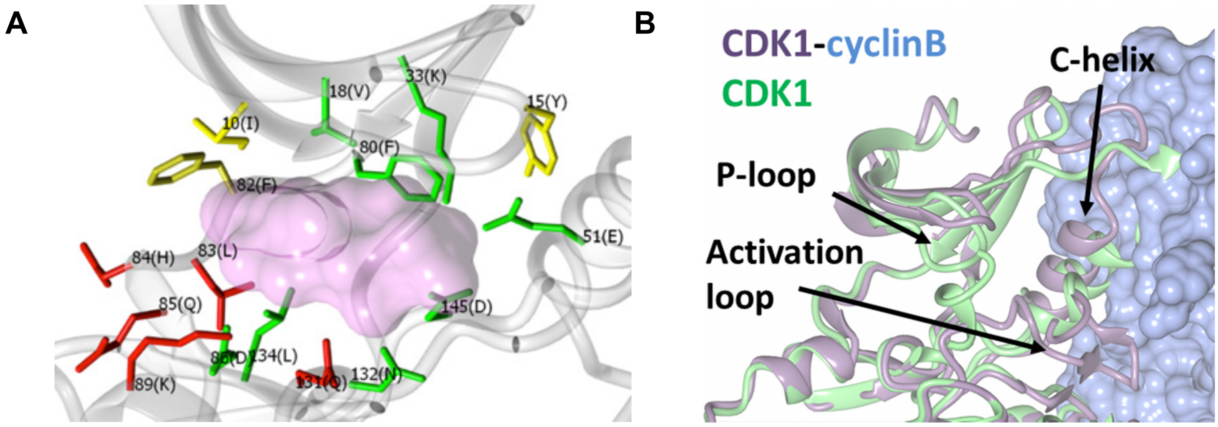

C

Residues numbers based on CDK2	10	15	18	33	51	80	82	83	84	85	86	89	131	132	134	145
CDK1	I	Y	V	K	E	F	F	L	S	M	D	K	Q	N	L	D
CDK2	I	Y	V	K	E	F	F	L	H	Q	D	K	Q	N	L	D
CDK4	I	Y	V	K	E	F	H	V	D	Q	D	T	E	N	L	D
CDK6	I	Y	V	K	E	F	H	V	D	Q	D	T	Q	N	L	D
CDK8	V	Y	V	K	E	F	Y	A	E	H	D	H	A	N	L	D
CDK9	I	F	V	K	E	F	F	C	E	H	D	G	A	N	L	D
CDK12	I	Y	V	K	E	F	Y	M	D	H	D	G	S	N	L	D

Figure S1. The conserved nature of the CDK catalytic site. Related to Figure 1. (*A*) The active site of CDK2-cyclin A (PDB entry 1FIN) with key active site residues rendered as cylinders, ATP drawn as a transparent molecular surface coloured pink. The overall fold is drawn in white ribbon representation. The CDK2 active site residues are coloured by conservation (green, conserved; yellow, similar, red, non-conserved) between human CDK1, CDK2, CDK4, CDK6, CDK8, CDK9, and CDK12 (Uniprot entries P06493, P24941, P11802, Q00534, P49336, P50750 and Q9NYV4 respectively). At the protein sequence level, full length CDK1 and CDK2 share 64.5% sequence identity and 77.6 % similarity (EMBOSS Needle, EMBL-EBI). (*B*) CDK1 in the two structures (PDB entries 4YC3 (CDK1-cyclinB-Cks2, where CDK1 is represented in lilac ribbon and cyclin B in blue surface) and 4YC6 (CDK1-Cks1, where CDK1 is represented in green ribbon) differ through ordering of the N-terminal lobe and activation loop upon binding of cyclin

2

B. (*C*) Alignment of CDKs and their active site residues depicted in A), coloured by conservation (green, conserved; yellow, similar, red, non-conserved).

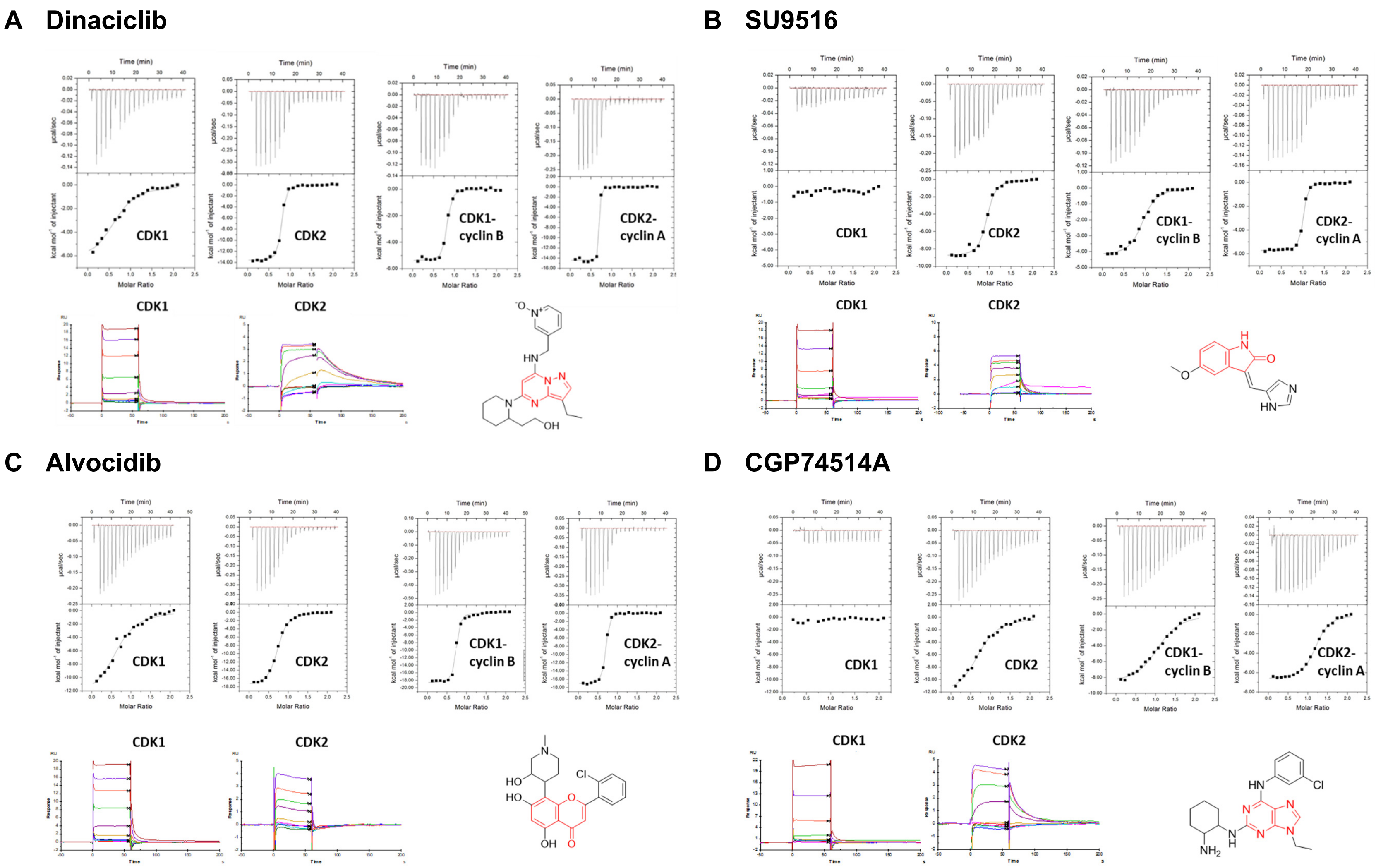
A Dinaciclib
B SU9516
C Alvocidib
D CGP74514A
Time (min)
µcal/sec
kcal mol⁻¹ of injectant
Molar Ratio
CDK1
CDK2
CDK1-
cyclin B
CDK2-
cyclin A
RU
Response
Time

Figure S2. ATP-competitive inhibitor binding to CDK1 and CDK2. Related to Table 1 and Figure 2. Isothermal titration calorimetry thermograms to assess binding of inhibitors Dinaciclib (*A*), SU9516 (*B*), Alvocidib (*C*), and CGP74514A (*D*) to CDK1, CDK2, CDK1-cyclin B, and CDK2- cyclin A. The inhibitors show reduced binding to monomeric CDK1 compared to CDK1-cyclin B (**Table 1**). Surface plasmon resonance sensorgrams to assess Dinaciclib (*A*), SU9516 (*B*), Alvocidib (*C*), and CGP74514A (*D*) binding to CDK1 and CDK2. Unphosphorylated CDK1 and CDK2 as GST fusions were immobilized on the SPR chip via anti-GST antibody coupling and inhibitor binding was assayed in duplicate over 11 (GST-CDK2) or 12 (GST-CDK1) concentrations as described in the Materials and Methods. Dissociation constants (**Table S2**) were derived by using the Biacore S200 Evaluation Software (GE Healthcare).

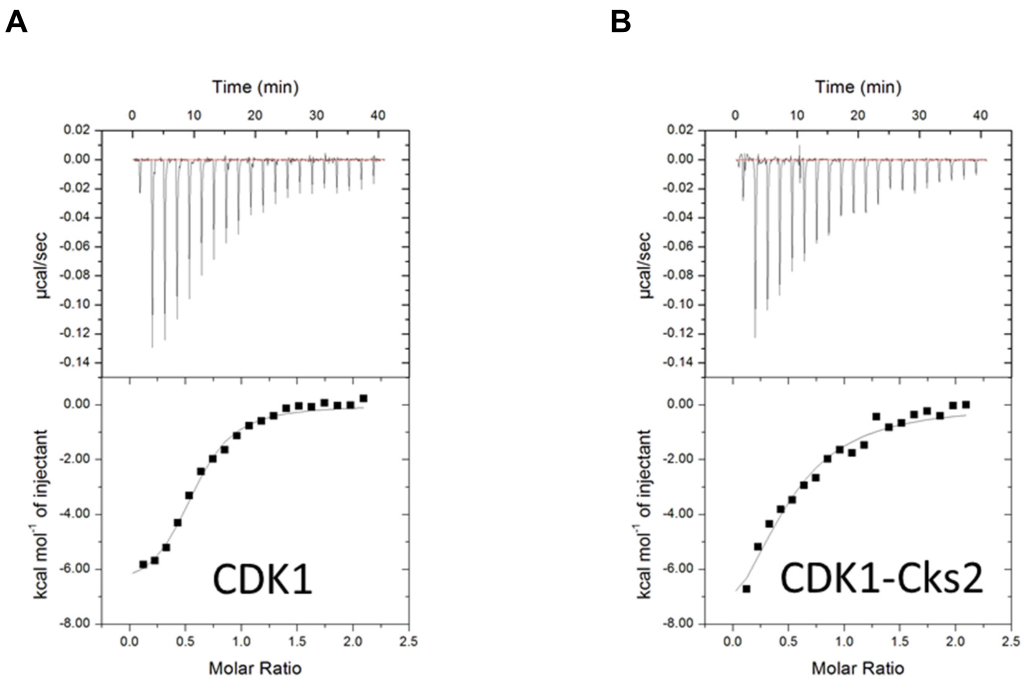

Figure S3. Comparison of the binding of Dinaciclib to CDK1 and CDK1-Cks2. Related to Figure 2. (*A*) Dinaciclib binding to CDK1. (This Figure panel is also included in Figure S1A). (*B*) Dinaciclib binding to CDK1-Cks2. This control was conducted in the presence of Cks2 so that any hypothesis inferred from the crystallographic observations would be supported. This titration experiment was performed as a singleton.

A CDK1

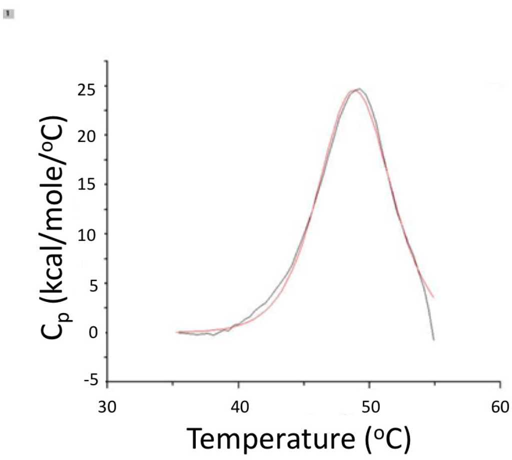

B CDK2

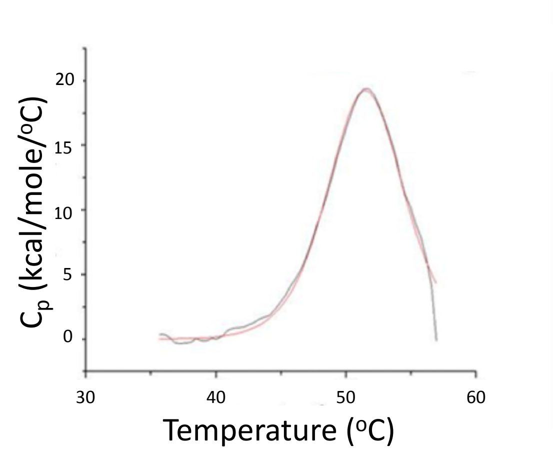

C CDK1

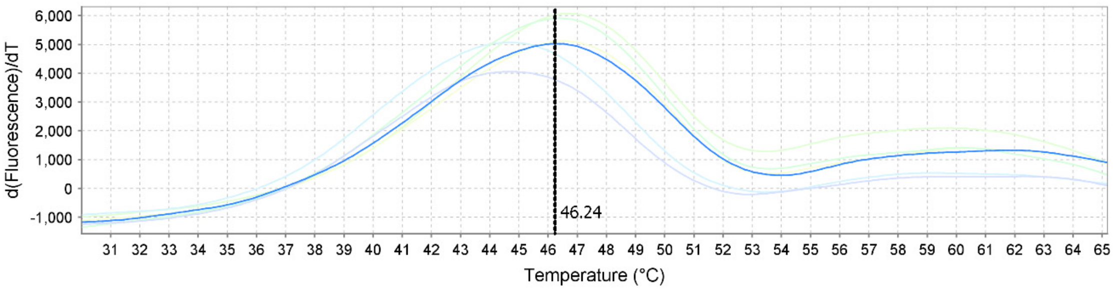

D CDK2

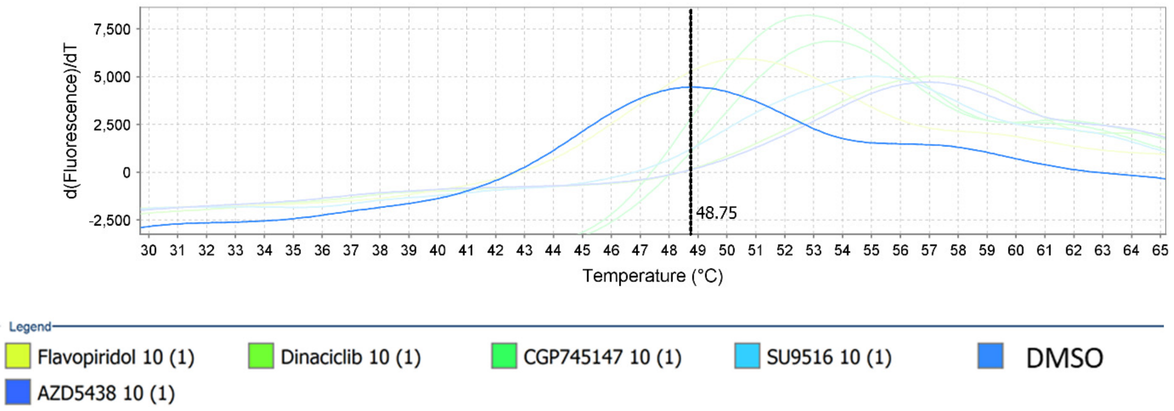

Figure S4. CDK1 and CDK2 stability assessed by differential scanning calorimetry and differential scanning fluorimetry Related to Table 2. (*A, B*) T_m determination. Differential

scanning calorimetry was used to determine T_m values for monomeric CDK1 and CDK2. (*C, D*) Differential scanning fluorimetry shows that ATP-competitive inhibitors have little effect on the stability of CDK1 (*C*) but stabilise CDK2 (*D*). Further experimental details are provided in the Materials and Methods.

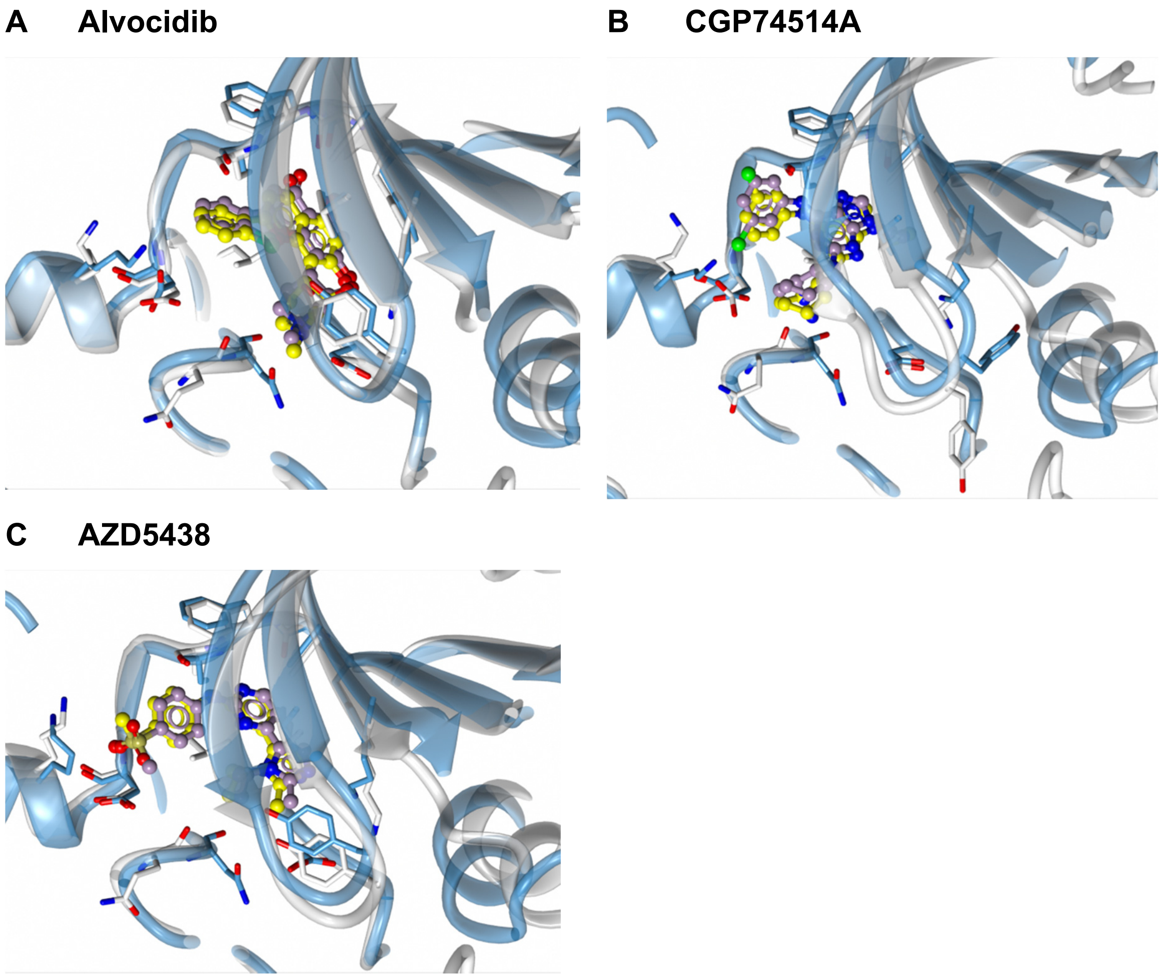

Figure S5. Comparison of ATP-competitive inhibitor binding to CDK1-cyclin B and CDK2-cyclin A. Related to Figure 3. Each of the three CDK1-cyclin B-Cks2-inhibitor co-complex structures presented in Figure 3 is overlaid with the corresponding CDK2-cyclin A-inhibitor co-complex structure. (*A*) Alvocidib, (*B*) CGP74514a and (*C*) AZD5438. The view in each case is identical to the view shown in Figure 3. CDK1-cyclin B is shown in white ribbon and cylinder with the inhibitor carbon atoms coloured yellow. CDK2-cyclin A is drawn in light blue with inhibitor carbon atoms coloured lilac.

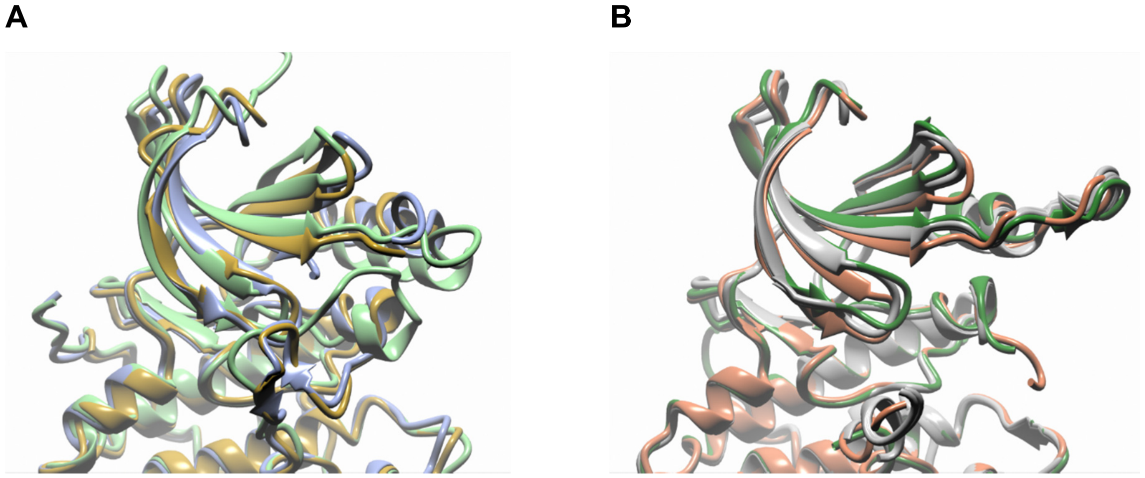

Figure S6. Comparison of cyclin-free CDK2 and CDK1 structural poses. Related to Figure 2-4 (*A*) Showing the flexibility of Cyclin-free CDK2 within the $P2_12_12_1$ crystal form; apo CDK2 (1HCK – blue), cyclin-free CDK2 bound to ANS (3PXZ - green), and the alternative lattice formed through binding of Cks1 (1BUH – gold) suggesting that this crystal form is capable of accommodating significantly different kinase conformations. (*B*) Superimposition of the 4 ASU copies of cyclin-free CDK1 (4YC6 - grey) with the two alternative lattices formed through association with dinaciclib (6GU6 - coral) and AZD5438 (6GU7 - green).

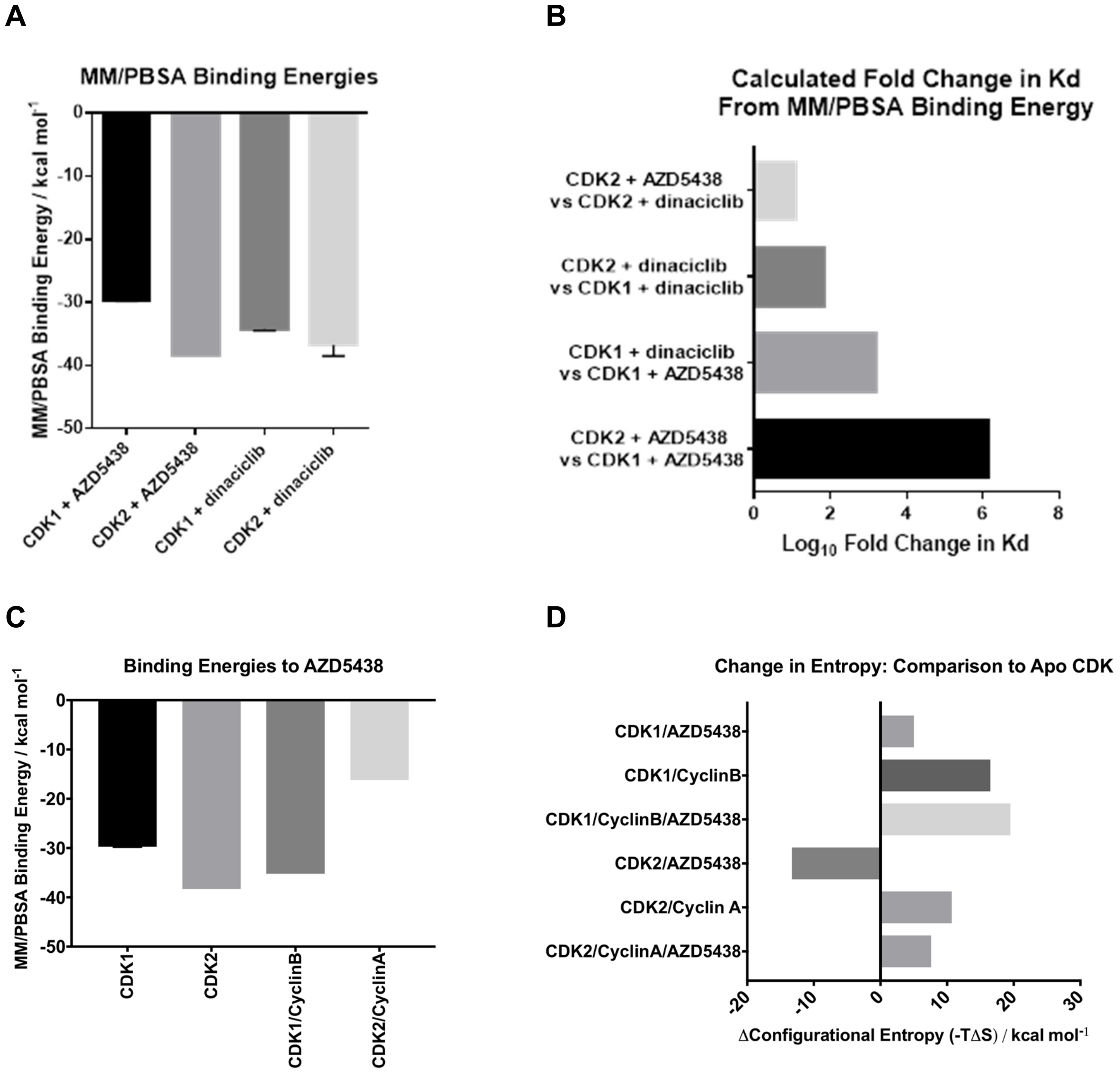

Figure S7. Simulations binding data for AZD5438. Related to Figure 2. In silico molecular dynamics simulations and MM/PBSA calculations against cyclin-free CDK1 and CDK2 with AZD5438 and Dinaciclib (A) and calculated fold change in Kd (B). Simulation analysis for monomeric and complex binding to AZD5438: binding energies derived from molecular dynamics simulations (C), and calculated change in configurational entropy in the CDK component of protein, ligand, and protein-ligand complexes (D).

SUPPLEMENTAL TABLES

	Published measurements (nM)		Lab measurements (nM)		Method	Reference
	CDK1B	CDK2A	CDK1B	CDK2A		
Dinaciclib	3/3/3/3	1/1/1/1	23 (12.8)	3.5 (1.9)	γ^{33}P-ATP streptavidin coated bead-based assay (biotinylated peptide of Histone H1)	(Whittaker et al., 2017), (Asghar et al., 2015), (Mariaule and Belmont, 2014), (Parry et al., 2010)
AZD5438	16/16	3/45	12 (6.7)	7.4 (4.1)	Scintillation proximity assay	(Whittaker et al., 2017), (Byth et al., 2009)
Alvocidib	27/30/27/ 30/30	405/170/40 5/100 70	8.8 (4.9)	26 (14.4)	Radiolabelled γ^{33}P-ATP kinase assay	(Whittaker et al., 2017),(Asghar et al., 2015),(Byth et al., 2009), (Mariaule and Belmont, 2014), (Kim et al., 2000)
CGP74514A	25/25	n/a	833 (463)	33 (18.3)	Radiolabelled γ33P-ATP kinase assay	(Imbach et al., 1999)
SU9516	40/40	22/22	186 (103)	38 (21)	Radiolabelled γ^{33}P-ATP kinase assay	(Lane et al., 2001)

Table S1. Inhibitor IC_{50} values. Related to Figure 2. IC_{50} values for each of the five inhibitors against CDK1-cyclin B and CDK2-cyclin A were determined using an ADPGlo^{TM} assay format (Brown et al., 2015) and compared to literature values. K_i values denoted in parenthesis

A

	AZD5438 vs CDK1-cyclin B	AZD5438 vs CDK2-cyclin A	AZD5438 vs CDK1	AZD5438 vs CDK2
**N (sites)**	0.58	0.68	0.65	0.70
**K_a (M^{-1})**	$1.76 \times 10^7 \pm 1.83 \times 10^6$	$3.95 \times 10^8 \pm 1.38 \times 10^8$	$8.42 \times 10^5 \pm 1.54 \times 10^5$	$3.38 \times 10^7 \pm 4.01 \times 10^6$
**K_d (nM)**	57.0 ± 5.9	2.50 ± 0.88	1200 ± 220	29.5 ± 3.5
**ΔH (kcal/mol)**	-12.80 ± 0.11	-13.40 ± 0.06	-6.44 ± 0.38	-16.20 ± 0.11
**ΔS (cal/mol/deg)**	-9.1	-4.7	5.9	-19.1

	CGP74514A vs CDK1-cyclin B	CGP74514A vs CDK2-cyclin A	CGP74514A vs CDK1	CGP74514A vs CDK2
**N (sites)**	1.01	1.01	No fitting	1.17
**K_a (M^{-1})**	$1.11 \times 10^6 \pm 3.09 \times 10^5$	$4.74 \times 10^6 \pm 4.69 \times 10^5$		$1.43 \times 10^6 \pm 2.72 \times 10^5$
**K_d (nM)**	900 ± 250	210 ± 21	>20000	700 ± 130
**ΔH (kcal/mol)**	-6.09 ± 0.34	-6.09 ± 0.07		-4.60 ± 0.14
**ΔS (cal/mol/deg)**	7.6	10.5		13.0

	Dinaciclib vs CDK1-cyclin B	Dinaciclib vs CDK2-cyclin A	Dinaciclib vs CDK1	Dinaciclib vs CDK2
**N (sites)**	0.71	0.70	0.57	0.82
**K_a (M^{-1})**	$3.4 \times 10^7 \pm 5.64 \times 10^6$	$3.19 \times 10^8 \pm 1.55 \times 10^8$	$1.41 \times 10^6 \pm 2.34 \times 10^5$	$1.82 \times 10^7 \pm 3.95 \times 10^6$
**K_d (nM)**	29.4 ± 4.9	3.1 ± 1.5	709 ± 120	55 ± 12
**ΔH (kcal/mol)**	-7.10 ± 0.07	-12.40 ± 0.08	-6.9 ± 0.3	-13.30 ± 0.21
**ΔS (cal/mol/deg)**	11.0	-2.1	5.3	-10.8

	SU9516 vs CDK1-cyclin B	SU9516 vs CDK2-cyclin A	SU9516 vs CDK1	SU9516 vs CDK2
**N (sites)**	0.97	0.98	No fitting	0.95
**K_a (M^{-1})**	$4.68 \times 10^6 \pm 4.11 \times 10^5$	$3.28 \times 10^7 \pm 3.06 \times 10^6$		$1.02 \times 10^7 \pm 1.57 \times 10^6$
**K_d (nM)**	214 ± 19	30.5 ± 2.8	>20000	98 ± 15
**ΔH (kcal/mol)**	-4.39 ± 0.04	-5.02 ± 0.03		-6.28 ± 0.08
**ΔS (cal/mol/deg)**	16.0	17.8		11.4

	Alvocidib vs CDK1-cyclin B	Alvocidib vs CDK2-cyclin A	Alvocidib vs CDK1	Alvocidib vs CDK2
**N (sites)**	0.71	0.80	0.61	0.82
**K_a (M^{-1})**	$4.51 \times 10^7 \pm 8.5 \times 10^6$	$5.08 \times 10^7 \pm 1.04 \times 10^7$	$5.15 \times 10^5 \pm 7.89 \times 10^4$	$4.6 \times 10^6 \pm 5.01 \times 10^5$
**K_d (nM)**	22.2 ± 4.2	20 ± 4	1900 ± 300	217.0 ± 23.7
**ΔH (kcal/mol)**	-16.60 ± 0.16	-15.10 ± 0.14	-10.70 ± 0.74	-13.80 ± 0.18
**ΔS (cal/mol/deg)**	-19.8	-14.5	-9.3	-14.9

B

	AZD5438 vs CDK1-cyclin B	AZD5438 vs CDK2-cyclin A	AZD5438 vs CDK1	AZD5438 vs CDK2
**N (sites)**	0.58	0.56	0.65	0.56
**K_a (M^{-1})**	$1.20 \times 10^7 \pm 3.77 \times 10^6$	$2.22 \times 10^8 \pm 4.73 \times 10^7$	$1.32 \times 10^5 \pm 2.53 \times 10^4$	$4.28 \times 10^7 \pm 3.71 \times 10^6$
**K_d (nM)**	83 ± 26	4.5 ± 1.0	7600 ± 1500	23 ± 2
**ΔH (kcal/mol)**	-14.13 ± 0.42	-16 ± 0.08	-18.8 ± 3.9	-19.9 ± 0.1
**ΔS (cal/mol/deg)**	-14.2	-14.6	-38.5	-30.7

	CGP74514A vs CDK1-cyclin B	CGP74514A vs CDK2-cyclin A	CGP74514A vs CDK1	CGP74514A vs CDK2
**N (sites)**	0.73	1.18	No fitting	1.09
**K_a (M^{-1})**	$9.82 \times 10^5 \pm 1.31 \times 10^5$	$4.21 \times 10^6 \pm 3.34 \times 10^5$		$1.37 \times 10^6 \pm 2.27 \times 10^5$
**K_d (nM)**	1000 ± 140	240 ± 19	>20000	730 ± 120
**ΔH (kcal/mol)**	-12.6 ± 0.5	-6.70 ± 0.06		-8.73 ± 0.25
**ΔS (cal/mol/deg)**	-14.1	8.2		-0.7

	Dinaciclib vs CDK1-cyclin B	Dinaciclib vs CDK2-cyclin A	Dinaciclib vs CDK1	Dinaciclib vs CDK2
**N (sites)**	0.77	0.65	0.67	0.75
**K_a (M^{-1})**	$2.93 \times 10^7 \pm 4.71 \times 10^6$	$9.93 \times 10^8 \pm 3.73 \times 10^8$	$8.44 \times 10^5 \pm 1.68 \times 10^5$	$3.73 \times 10^7 \pm 3.74 \times 10^6$
**K_d (nM)**	34.0 ± 5.5	1.0 ± 0.4	1200 ± 240	27 ± 3
**ΔH (kcal/mol)**	-5.40 ± 0.05	-14.40 ± 0.09	-6.56 ± 0.39	-13.70 ± 0.08
**ΔS (cal/mol/deg)**	16.4	-6.43	5.5	-10.4

	SU9516 vs CDK1-cyclin B	SU9516 vs CDK2-cyclin A	SU9516 vs CDK1	SU9516 vs CDK2
**N (sites)**	0.91	0.96	No fitting	0.90
**K_a (M^{-1})**	$3.94 \times 10^6 \pm 5.53 \times 10^5$	$4.88 \times 10^7 \pm 4.1 \times 10^6$		$1.02 \times 10^7 \pm 1.77 \times 10^6$
**K_d (nM)**	254 ± 36	21.0 ± 1.7	>20000	98 ± 17
**ΔH (kcal/mol)**	-4.24 ± 0.07	-5.72 ± 0.02		-8.81 ± 0.13
**ΔS (cal/mol/deg)**	16.2	16.3		3.0

	Alvocidib vs CDK1-cyclin B	Alvocidib vs CDK2-cyclin A	Alvocidib vs CDK1	Alvocidib vs CDK2
**N (sites)**	0.69	0.65	0.68	0.70
**K_a (M^{-1})**	$3.01 \times 10^7 \pm 6.5 \times 10^6$	$4 \times 10^7 \pm 3.1 \times 10^6$	$7.44 \times 10^5 \pm 1.92 \times 10^5$	$4.63 \times 10^6 \pm 2.08 \times 10^5$
**K_d (nM)**	33.0 ± 7.2	25 ± 2	1300 ± 350	220.0 ± 9.7
**ΔH (kcal/mol)**	-18.60 ± 0.24	-17.10 ± 0.08	-13.2 ± 1.1	-17.8 ± 0.1
**ΔS (cal/mol/deg)**	-27.3	-21.5	-16.6	-28.2

Table S2. ITC experimental data for inhibitor binding to cyclin-free CDK1, CDK2 and their respective complexes with cognate cyclin partners. Related to Figure 2. Experiments were carried out in duplicate biological replicates shown in panels A and B.

Table S3. Summary of data collection and structure refinement. related to Figures 3-5

See associated Excel file: 'TableS3CrystallographyData.xlsx'
